# Engineering of Isogenic Cells Deficient for MR1 with a CRISPR/Cas9 Lentiviral System: Tools To Study Microbial Antigen Processing and Presentation to Human MR1-Restricted T Cells

**DOI:** 10.4049/jimmunol.1501402

**Published:** 2016-06-15

**Authors:** Bruno Laugel, Angharad Lloyd, Erin W. Meermeier, Michael D. Crowther, Thomas R. Connor, Garry Dolton, John J. Miles, Scott R. Burrows, Marielle C. Gold, David M. Lewinsohn, Andrew K. Sewell

**Affiliations:** * Division of Infection and Immunity, Cardiff University School of Medicine, Heath Park, Cardiff CF14 4XN, United Kingdom;; † Department of Pulmonary and Critical Care Medicine, Oregon Health and Science University, Portland, OR 97239;; ‡ Cardiff School of Biosciences, Cardiff University, Cardiff CF10 3AX, United Kingdom; and; § QIMR Berghofer Medical Research Institute, Brisbane, Queensland 4006, Australia

## Abstract

The nonclassical HLA molecule MHC-related protein 1 (MR1) presents metabolites of the vitamin B synthesis pathways to mucosal-associated invariant T (MAIT) cells and other MR1-restricted T cells. This new class of Ags represents a variation on the classical paradigm of self/non-self discrimination because these T cells are activated through their TCR by small organic compounds generated during microbial vitamin B_2_ synthesis. Beyond the fundamental significance, the invariant nature of MR1 across the human population is a tantalizing feature for the potential development of universal immune therapeutic and diagnostic tools. However, many aspects of MR1 Ag presentation and MR1-restricted T cell biology remain unknown, and the ubiquitous expression of MR1 across tissues and cell lines can be a confounding factor for experimental purposes. In this study, we report the development of a novel CRISPR/Cas9 genome editing lentiviral system and its use to efficiently disrupt MR1 expression in A459, THP-1, and K562 cell lines. We generated isogenic MR1^−/−^ clonal derivatives of the A549 lung carcinoma and THP-1 monocytic cell lines and used these to study T cell responses to intracellular pathogens. We confirmed that MAIT cell clones were unable to respond to MR1^−/−^ clones infected with bacteria whereas Ag presentation by classical and other nonclassical HLAs was unaffected. This system represents a robust and efficient method to disrupt the expression of MR1 and should facilitate investigations into the processing and presentation of MR1 Ags as well as into the biology of MAIT cells.

## Introduction

Mucosal-associated invariant T (MAIT) cells are the most abundant nonconventional T cell subset, accounting for up to 5% of all T cells in humans, and are thought to be important for the control of a number of bacterial, fungal, and yeast infections (1–5). These so-called innate-like T cells, which are mostly found in the blood, the liver, and at mucosal surfaces, express a semi-invariant TCR consisting of an α-chain using the canonical TRAV1-2–TRAJ33/12/20 (Vα7.2-Jα33/12/20) rearrangements (6). MAIT cells acquire effector functions during thymic selection and readily respond to Ags derived from many (but not all) bacteria such as *Escherichia coli*, *Klebsiella pneumoniae*, *Mycobacterium tuberculosis*, or *Staphylococcus epidermis* as well as several yeast species in the periphery without prior priming (3, 7). MAIT cell activation is mediated by the interaction between the TCR and microbe-derived Ags presented by the nonclassical MHC-related protein 1 (MR1) and results in the secretion of cytokines as well as in granzyme- and perforin-dependent cytoxicity (2, 8). The nature of these Ags has been recently discovered by Kjer-Nielsen et al. (9) who showed that MR1 binds and presents small organic metabolite compounds derived from the vitamin B synthesis pathways (10). A number of intermediates of the folic acid (vitamin B_9_) and riboflavin (vitamin B_2_) pathways act as ligands for MR1 (10, 11). However, only compounds derived from the riboflavin pathway, which is absent in mammals but present in microbes, were found to activate MAIT cells, therefore providing a molecular basis for the specific recognition of microbially infected cells (9). Our recent study showed that human MAIT cells isolated from a single individual use distinct TCR repertoires to recognize cells infected with different bacteria in an MR1-specific manner (12). Moreover, Gherardin et al. (13) have recently characterized the crystal structure and biophysical properties of TCRs from T cells with discrete Ag specificity for folate- or riboflavin-derived compounds presented by MR1. Remarkably, several of these MR1-restricted T cell clonotypes did not express the canonical MAIT TRAV1-2 TCR α-chain (13), indicating that non-MAIT αβ T cells are also able to recognize MR1 Ags. This TCR usage heterogeneity may provide a degree of specificity in MAIT- and MR1-restricted T cell activation and hints that different pathogens could generate MR1-restricted Ags of varied structure and chemical composition. In addition to MR1-restricted activation, MAIT cells respond to proinflammatory innate cytokines such as IL-12 and IL-18 (1, 14), which can act as autonomous stimuli or combine with TCR signals to potentiate MAIT cell activation (15). This Ag-independent activation process may be relevant to the pathogenesis of a number of inflammatory conditions in which the number, distribution, phenotype, and functions of MAIT cells were found to be altered (1, 16–18).

The biology of MR1-restricted T cells is a rapidly emerging field in immunology. The invariant nature of MR1 across the human population and its established role in the presentation of pathogen-derived Ags are of outstanding interest for the potential development of universal therapeutic and diagnostic tools in infectious diseases. MR1 expression also appears to be ubiquitous among different cells and tissues (19, 20), which may indicate that MR1-driven Ag responses are relevant to the pathogenesis of a broad number of immune-mediated diseases. However, the invariance and ubiquity of MR1 also complicate basic investigations of its ligand-binding and Ag presentation properties as well as in the understanding of MR1-restricted T cell biology. Indeed, the presence of MR1 on most APC lines and primary cells that also express other classical and nonclassical HLA molecules can make the unambiguous identification of microbe-specific MAIT cells and their distinction from conventional T cells that express the TRAV1-2 TCR chain problematic. Besides, solely relying on known MAIT cell phenotypic markers could result in the exclusion of previously undescribed bona fide MR1-restricted T cells. So far, confirmation of MR1 restriction has exclusively relied on the use of an anti-MR1 blocking mAb to abrogate activation (21), yet this type of functional validation can sometimes produce inconclusive experimental data because of incomplete blockade. In this context, the availability of isogenic model cell lines bearing MR1 knockout mutations would be a valuable tool for reverse genetics and loss of function studies.

The clustered regularly interspaced palindromic repeats (CRISPR) and CRISPR-associated protein 9 (Cas9) genome editing platform has recently been developed as a powerful tool to induce targeted disruptive mutations in, or edit the DNA sequence of, a specific gene (22–28). Current CRISPR/Cas9 genome editing approaches rely on targeting the endonuclease activity of *Streptococcus pyogenes* or *Staphylococcus aureus* (29) Cas9 to a 17- to 23-nt-long genomic DNA sequence using a single-guide RNA (sgRNA) polynucleotide that acts both as a scaffold for Cas9 and as a DNA tethering agent. Cas9 induces DNA strand breaks near a so-called proto-spacer–associated motif (PAM) that are repaired either via nonhomologous end joining, often introducing insertions or deletions that disrupt the translational reading frame, or via homologous recombination using a repair template (25). CRISPR/Cas9 has been used to rapidly generate transgenic animals, correct deleterious mutations causing congenital diseases in cell lines and pluripotent stem cells, or identify and validate therapeutic targets. In this study, we report the generation of a novel versatile all-in-one CRISPR/Cas9 lentiviral vector and its use to derive isogenic clonal variants of carcinomic human alveolar basal epithelial A549 cells and of leukemic monocytic THP-1 cells commonly used to study the immune response to several intracellular bacteria, including *M. tuberculosis* and *Mycobacterium smegmatis* (2).

## Materials and Methods

### Generation of an all-in-one CRISPR/Cas9 lentivector

A synthetic polynucleotide sequence containing the canonical U6 RNA polymerase III promoter and an sgRNA sequence containing the *trans*-activating CRISPR RNA and the MR1-specific target sequence, based on the design of Mali et al. (24), was purchased from Eurofins MWG Operon (Ebersberg, Germany) and introduced in pCDNA.3-TOPO_wt-Cas9 (Addgene no. 41815) by nondirectional cloning at the unique Spe1 restriction site immediately upstream of the CMV promoter. To generate the all-in-one lentivector construct, the plasmid region containing the U6 promoter–sgRNA as well as the CMV promoter and Cas9 sequences was PCR amplified with primers containing Age1 and NsiI (pCDNA.3_Fwd and pCDNA.3_Rev) restriction sites from the pCDNA.3-TOPO_U6-sgRNA_wt-Cas9. This fragment was fused to a PCR amplicon consisting of the second generation pRRL.sin.cppt.pgk-gfp.wpre lentivector backbone developed by Didier Trono’s laboratory (Addgene no. 12252) devoid of the human PGK promoter and GFP cDNA. This amplicon was obtained by PCR amplification with primers containing matching restriction sites (pRRL.0_Fwd and pRRL.0_Rev). The final sequence of the resulting pRRL.sin.CRISPR/Cas9 plasmid and all the primer sequences are provided in Supplemental Fig. 1 and Supplemental Table I, respectively.

### Introduction of novel sgRNA target sequences

Repurposing of the CRISPR/Cas9 system was performed by introducing new 19-nt target sequences using a PCR cloning approach whereby the new sequence is fused to 5′-phosphate–modified primers complementary to plasmid sequences immediately flanking the target sequence (forward primer. 5′-GTTTTAGAGCTAGAAATAGCAAGTTAAx_9_-3′, reverse primer, 5′-GGTGTTTCGTCCTTTCCx_10_-3′, where x represents nucleotides of the target sequence). PCR amplification conditions were as follows: an initial 2-min denaturation step at 95°C followed by 30 three-step cycles (30 s denaturation at 95°C, 30 s annealing at 53°C, 5 min elongation at 72°C) and a final elongation step of 5 min at 72°C using the Phusion high-fidelity DNA polymerase (Thermo Scientific, Cambridge, U.K.). The PCR product corresponding to the full-size plasmid (∼11 kbp) was gel extracted, purified, and circularized by ligation with standard T4 DNA ligase (Promega, Southampton, U.K.). Ligation products were then transformed into *Escherichia coli* XL10 Gold (Stratagene, La Jolla, CA). Bacteria were plated onto Luria–Bertani agar containing 100 μg/ml carbenicillin (Biochemical Direct) following a 1-h recovery step at 37°C in SOC medium (Invitrogen, Paisley, U.K.). After an overnight incubation at 37°C, 10 colonies per new target sequence were tested by colony PCR to assess plasmid integrity and confirm the presence of the sgRNA region of interest (forward primer pLKO1.A; reverse primer gRNAcolPCR.R). PCR products of the expected size (450 bp) were purified, cleaned on DNA Clean and Concentrator columns (Zymo Research, Irvine, CA), and sent for sequencing. A total of five sgRNA target sequences were introduced for testing: one targeting exon 1, three targeting exon 2, and one specific for a site on exon 3 (Table I).

### Cell culture and clonal expansion of MR1^−/−^ cells

Adenocarcinoma HeLa (ATCC CCL-2), human acute monocytic leukemia THP-1 (ATCC TIB-202), lymphoblastic chronic myelogenous leukemic K-562 (ATCC CCL-243), and carcinomic alveolar basal epithelial A549 (ATCC CCL-185) cell lines were maintained in RPMI 1640 growth medium (Life Technologies, Paisley, U.K.) supplemented with 2 mM glutamine, 50 U/ml penicillin, and 50 μg/ml streptomycin (Life Technologies) and 10% FBS (Life Technologies), referred to as R10 hereon. Cells were passaged when reaching 80% confluence using enzyme-free cell-dissociation buffer HBSS (Life Technologies). Bulk A549 cells transfected with the CRISPR/Cas9 plasmid were sorted by flow cytometry by gating on the MR1^−^ population. MR1^−/−^ cells were isolated from bulk THP-1 cells transduced with the CRISPR/Cas9 lentivector and subjected to MAIT cell positive selection. The selection was performed by using THP-1 cells infected with *M. smegmatis* at a 20:1 multiplicity of infection. Target cells were cocultured for 7 d with the MAIT cell clone D481A9 at an E:T ratio of 1:2. After the selection period, cells were transferred to flasks for a larger cell culture and further expanded. Subsequently, the resulting cells were clonally expanded by isolating single cells using a limiting dilution approach. An average of 0.3 cell was plated in individual wells of four flat-bottom 96-well plates containing 200 μl medium. A total of 16 clonal A549 and 20 THP-1 populations were then screened by flow cytometry. Three A549 and five THP-1 clones were ultimately selected and analyzed using a mismatch-specific endonuclease assay as described in *Results*. T cell clones were cultured in RPMI 1640 media supplemented with 2 mM glutamine, 50 U/ml penicillin, and 50 μg/ml streptomycin (Life Technologies), 10% FBS, 0.01 M HEPES buffer, nonessential amino acids, sodium pyruvate (Life Technologies), 25 ng/ml IL-15 (PeproTech, Rocky Hill, NJ), and either 20 or 200 IU/ml IL-2 (aldesleukin [Proleukin]; Prometheus, San Diego, CA), depending on the stage of culture.

### MAIT and T cell clones activation

IFN-γ ELISPOT assays with the HLA-E–restricted T cell clone D160 1–23 were performed as described in Lewinsohn et al. (30). Briefly, A549 cells or MR1^−/−^ A549 cells (10^4^/well) were incubated with 5 mg/ml *M. tuberculosis* pronase-digested cell wall (31) in RPMI 1640 medium supplemented with 10% human serum for 2 h. D160 1–23 (10^4^/well) was then added to each well. The plate was incubated overnight at 37°C and then developed using an AEC Vectastain kit (Vector Laboratories). PHA stimulation was used as a positive control for T cell signaling viability. Data shown are representative of three independent experiments. The D426B1 and D481 MAIT cell clones were washed with RPMI 1640 medium and then rested in RPMI 1640 supplemented with 50 U/ml penicillin and 50 μg/ml streptomycin, 2 mM l-glutamine, and 5% FBS overnight. A549 cells (wild-type [WT] and MR1 knockouts) were cultured in antibiotic-free R10 overnight. A549 and THP-1 cells were exposed to *M. smegmatis* at a multiplicity of infection of 100:1 (bacteria to cells) for 2 h in antibiotic-free R10, followed by 2 h incubation with R10 containing 50 U/ml penicillin and 50 μg/ml streptomycin (Life Technologies). A549 and THP-1 cells were washed to remove extracellular *M. smegmatis*. Control cells were mock treated as if they had been coincubated with bacteria. A549 and THP-1 cells were then plated into 96U-well plates at a density of 6 × 10^4^ cells per well. MAIT cells (3 × 10^4^) were then added to each well and cultured overnight. Supernatant (60 μl) was then harvested, diluted to 120 μl with R5, and assayed for MIP-1β, TNF-α, and IFN-γ ELISA (R&D Systems) according to the manufacturer’s instructions. Data were plotted and analyzed with GraphPad Prism software version 5.03. Activation of the CD8^+^ T cell clone B9 specific for an octameric peptide (SELEIKRY) derived from the EBV BZLF1 protein and presented by HLA-B*1801 (32) was done by coculturing T cells with A549 WT or MR1^−/−^ cells overnight in 200 μl R5. A549 cells (1 × 10^6^) were pulsed with each peptide concentration in 100 μl R5 for 2 h at 37°C. The cells were washed with 10 ml R5 five times, aspirating all excess supernatant between washes. The A549 cells were counted and resuspended at 6 × 10^5^ cells/ml in a final volume of 700 μl. Then, 100 μl cell suspension (i.e., 60,000 cells) was plated into 96-well plates. T cells (3 × 10^4^) in 100 μl R5 were added into each well. Then, the remaining APCs were centrifuged and the supernatant was plated with T cells as a control to ensure that the T cell response was mediated through APC presentation and not T:T presentation. Each assay condition was performed in triplicate. For ELISAs, 50 μl supernatant per well was used for TNF-α and IFN-γ ELISA and 25 μl supernatant diluted with 25 μl of R5 per well was used for MIP-1β. Supernatants were frozen and assayed for MIP-1β and IFN-γ in a sandwich ELISA assay (R&D Systems) according to the manufacturer’s instructions.

### Flow cytometry

The PE-conjugated anti-MR1 Ab clone 26.5 (BioLegend, London, U.K.) was used to stain HeLa-MR1, A549, and THP-1 cells at the dilution recommended by the manufacturer. To stabilize the cell-surface levels of MR1, A549 cells were incubated overnight in the presence of 50 μg/ml acetyl-6-formylpterine (Schircks Laboratories, Jona, Switzerland) prior to staining (11). Where indicated, the W6/32 monoclonal anti–HLA-ABC Ab conjugated to APC (eBioscience, Hatfield, U.K.) was also used to monitor classical HLA-I levels on the cell surface. 7-Aminoactinomycin D (BD Biosciences, Oxford, U.K.) or the viability Vivid Dye (Molecular Probes, Life Technologies) was added to all the staining before data acquisition on a FACSCalibur instrument (Becton-Dickinson, UK) or a FACSCanto II (Becton Dickinson, Oxford, U.K.). Data analysis was performed with FlowJo software (Tree Star, Ashland, OR) by drawing a population gate on the forward scatter area/side scatter area plot and on viable cells (7-aminoactinomycin D^−^ or Vivid^−^).

### Monitoring of mutations at the MR1 locus

Genomic DNA from A549 and THP-1 cells was isolated with the GenElute mammalian genomic DNA miniprep kit (Sigma-Aldrich, Gillingham, U.K.). Mutations at the target site were detected using the CEL-I enzyme, as part of the Surveyor assay (Transgenomic, Glasgow, U.K.), which cleaves DNA duplexes bearing base pair mismatches, caused by insertions or deletions at proximity of the PAM sequence, within the PCR amplicons generated with primers flanking the genomic target site. The PCR forward primer (SURV1_Fwd) is located in the intron region upstream of the target site, and the reverse primer (SURV1_Rev) is located downstream on exon 2. The predicted size of the full-length PCR product was 852 bp, and the expected position of Cas9 cleavage (immediately downstream of the PAM sequence) is located 521 bp downstream of the start of the forward primer and 331 bp from the reverse primer. In the case of HeLa-MR1 cells, which overexpress MR1 cDNA, a PCR amplicon flanking the sgRNA target sequences was obtained by amplifying a 360-bp region from template DNA isolated as described above using a forward primer complementary to a plasmid region immediately upstream of the MR1 cDNA (SURV2_Fwd) and a reverse primer downstream of the target site (SURV2_Rev). To sequence the modified MR1 locus in the A549 clonal derivatives clone 9 and clone 11, the same primers fused to the type IIs endonuclease BsaI were used to amplify the genomic DNA and clone the amplicons into a cloning vector (Addgene no. 32189). Plasmid minipreps from 10 colonies obtained from the resulting transformation for each clone were sent for Sanger sequencing at Eurofins MWG Operon.

### Identification of CRISPR/Cas9 mutagenesis off-target effects using genomics

Undertaking a BLAST search against the human genome refseq database using the guide RNA (gRNA) sequence, we identified six sites (excluding MR1) located within genes that contained sequence that differed at four or fewer sites compared with the gRNA. Having identified these genes, we undertook whole-genome sequence of two samples, clone 9 and clone 11, to assess whether these genes contained off-target effects. To sequence the sample, we extracted the genomic DNA from the two A549 clones using the GenElute mammalian genomic DNA miniprep kit (Sigma-Aldrich). We fragmented 1 μg DNA to an average of 300 bp fragments by sonication and prepared libraries using NEBNext ultra library preparation kits (New England Biolabs, Herts, U.K.). The libraries were sequenced using a NextSeq 500, running high output 150-bp paired end sequencing to a depth of >20× coverage for each genome. Taking the sequence reads generated, we mapped these against the gene sequences of the target sites, and in five of the six cases using the Burrows–Wheeler alignment tool (33), visualizing the mapping results using Artemis (34). To identify inserts that are larger than those that could be detected by mapping, we screened all of the reads generated using the sequence 20 bp on the 5′ side of each of the off-target sites identified above. Extracting these reads, we identified any cases where larger deletions were present within these reads by manually examining the sequence of the reads by eye against the expected reference sequence. Following this we also undertook an analysis to identify other potential off-target sites that may not have been in genes, or detected by our screen of the human genome. To do this, we identified all of the sequence reads that contained a sequence matching, or complementary to, the gRNA at 15 of 20 bases, and extracted these into a set of fastq files. We then assembled these sequences using Velvet and extracted all contigs with a sequence length of >200 bp. In total, this analysis identified 7574 locations in sample clone 9 and 7516 locations in clone 11 for further investigation. Extracting the BLAST alignments for each of the sequences queried, we examined, by eye, all of the alignments containing insertions within the contig for evidence of insertions within 100 bp of the match to the gRNA. In total, other than the known deletion within MR1 and the detected RP11-46A10.6 site, we found no other cases where there appeared to have been an off-target effect in either of our samples. Sequencing data were submitted to the EMBL-EBI European Nucleotide Archive (http://www.ebi.ac.uk/ena). The study accession number is PRJEB12991. Sample accession and secondary accession numbers for A549 clone 9 are ERS1078785 and SAMEA3891651. Sample accession and secondary accession numbers for A549 clone 11 are ERS1078786 and SAMEA3891652. Experiment accession and run accession numbers for A549 clone 9 are ERX1378672 and ERR1307049. Experiment accession and run accession numbers for A549 clone 11 are ERX1378673 and ERR1307050.

### Production of lentiviral particles and cell transduction

The pRRL.sin.CRISPR/Cas9 lentivector was cotransfected with the second generation VSV.G envelope pMD2.G (Addgene no. 12259) and packaging pCMV-dR8.74 (Addgene no. 22036) plasmids in HEK-293T cells by CaCl_2_ transfection in six-well plates. Lentiviral supernatants (∼10 ml total volume) were collected at 24 and 48 h, filtered using a 0.45-μm cellulose acetate syringe filter, concentrated by centrifugation at 28,000 rpm for 2 h, and resuspended in 0.5 ml R10. HeLa-MR1, THP-1, K562, or A549 cells grown to ∼50% confluence in six-well plates were transduced with the concentrated viral supernatant diluted 3-fold in a final volume of 3 ml.

## Results

### Development of a versatile all-in-one lentiviral CRISPR/Cas9 system

**FIGURE 1.**
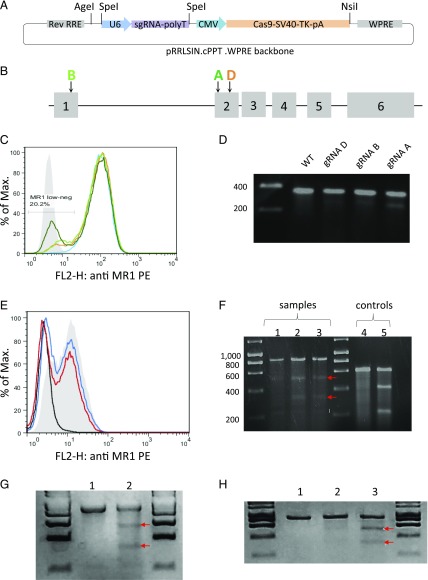
Description of the lentiviral CRISPR/Cas9 system and identification of gRNA target sequences efficiently disrupting MR1 gene expression. (**A**) Graphic representation of the all-in-one lentiviral system generated for this study. The CRISPR/Cas9 elements derived from the pCDNA.3-TOPO_wt-Cas9 plasmid were incorporated within the second generation pRRLSIN.cPPT.WPRE lentivector backbone between the Age1 and Nsi1 unique restriction sites. (**B**) Positioning of three active sgRNA target sequences with respect to the intron/exon structure of the MR1 gene. (**C**) Disruption of MR1 protein surface expression on HeLa reporter cells overexpressing MR1 transduced with lentiviruses expressing Cas9 in concert with a control gRNA (light blue histogram), MR1 gRNAs A (dark green histogram), B (light green histogram), or D (orange histogram). Isotype control staining is shown as a solid light gray histogram. The histogram plot shown is representative of four independent staining experiments. (**D**) Monitoring of mutations within the genomic DNA of transduced HeLa cells. MR1 cDNA PCR amplicons generated with the genomic DNA of WT cells or of cells transduced with lentiviruses expressing each sgRNA target were denatured, annealed, and digested with the CEL-I enzyme as described in *Materials and Methods*. Four hundred (WT lane) or 200 ng of PCR products from unmodified HeLa-MR1 cells mixed with 200 ng amplicon generated with DNA obtained from cells transduced with each sgRNA target (gRNA D, B, and A lanes) were used. Digestion of PCR amplicons was only obvious for the WT/gRNA A heteroduplexes (lane gRNA A). Data shown are representative of two independent experiments. (**E**) A549 cells were lipofected with plasmid DNA containing the CRISPR/Cas9 elements (including the gRNA A target sequence) or transduced with lentiviral particles made from the same plasmid. Eight days after transduction/lipofection, cells were treated overnight with acetyl-6-formylpterine (50 μg/ml) prior to flow cytometry analysis. A549 cells stained with an isotype control Ab are shown as a black histogram (MFI = 2.78), mock lipofected cells as a solid gray histogram (MFI = 9.53), cells transduced with lentivirus as a red histogram (MFI = 5.84), and those lipofected with the CRISPR/Cas9 plasmid DNA as a blue histogram (MFI = 7.07). The data shown are representative of three stainings. (**F**) Surveyor assay performed with a total of 500 ng PCR amplicon obtained from genomic DNA of unmodified A549 cells or from cells lipofected or transduced with gRNA.A CRISPR/Cas9. Homoduplex WT DNA (*lane 1*) or heteroduplexes of annealed WT and modified amplicons from transduced cells (*lane 2*) or lipofected cells (*lane 3*) were digested with the CEL-I DNA-mismatch–specific enzyme. Two bands matching the sizes of the expected digestion products (521 and 331 bp) can be identified (indicated with red arrows). Undigested (*lane 4*) and digested (*lane 5*) assay controls are shown. The gel shown is representative of at least five different experiments. (**G**) Surveyor assay performed with a total of 500 ng PCR amplicon obtained from genomic DNA of unmodified K562 cells or from cells transduced with gRNA.A CRISPR/Cas9. Homoduplex WT DNA (*lane 1*) or heteroduplexes of annealed WT and modified amplicons from transduced cells (*lane 2*) were digested with the CEL-I DNA-mismatch–specific enzyme. Two bands matching the sizes of the expected digestion products (521 and 331 bp) can be identified (indicated with red arrows). (**H**) Surveyor assay performed with a total of 500 ng PCR amplicon obtained from genomic DNA of unmodified THP-1 cells or from cells transduced with gRNA.A CRISPR/Cas9. Homoduplex WT DNA (*lane 1*) or heteroduplexes of annealed WT and modified amplicons from transduced cells before and after MAIT cell selection (*lanes 2* and *3*) were digested with the CEL-I DNA-mismatch–specific enzyme. Two bands matching the sizes of the expected digestion products (521 and 331 bp) can be identified (indicated with red arrows). The data shown in Fig. 1G and 1H are representative of two independent experiments.

We chose to adapt the two plasmid delivery platform described by Mali et al. (24), whereby the Cas9 and sgRNA elements are expressed on separate plasmids, to generate a single delivery system embedded within a second generation lentivector backbone. We reasoned that such a tool would combine the ease of an all-in-one delivery method with the high infectivity and broad cellular tropism of lentiviruses. For this purpose, we introduced a codon-optimized version of *S. pyogenes* Cas9 under the control of the CMV promoter as well as a U6 RNA polymerase III promoter sgRNA complex within the self-inactivating second generation lentivirus pRRL.sin.cppt.pgk-gfp.wpre (Fig. 1A), a well-characterized lentiviral system that produces high-titer viral particles. We designed the sgRNA so that the 19-nt target sequence could be easily swapped through a cloning PCR approach, a feature that distinguishes our lentivector from other available CRISPR/Cas9 systems.

### Generation of cell populations bearing CRISPR/Cas9-induced mutations in the MR1 locus

A total of five lentivectors, each containing a different CRISPR target sequence specific for the MR1 gene (Fig. 1B, Table I), were generated and tested in a reporter system consisting of HeLa cells overexpressing native human MR1 cDNA. MR1^low/−^ cells could be identified by flow cytometry within HeLa-MR1 cell populations transduced with three of the five lentiviruses (Fig. 1C). gRNA.A showed the highest disruption efficiency both at the protein and DNA levels (Fig. 1C, 1D). Based on these results, we selected gRNA.A to edit the genome of the A549 cell line. We compared MR1 gene modifications induced in A549 cells in which Cas9 and the MR1 gRNA.A were either stably or transiently expressed using lentiviral transduction or plasmid DNA transfection, respectively. Flow cytometry analysis showed that for both transient and stable CRISPR/Cas9 expression, the overall mean fluorescence intensities (MFIs) with anti-MR1 Ab were decreased and the proportion of cells appearing MR1 low or negative was increased compared with the control WT A549s (Fig. 1E). At the DNA level, the Surveyor assay revealed that DNA mismatches were introduced at the expected positions, as digestion of heteroduplex PCR amplicons containing the CRISPR/Cas9 target sites from WT and modified cells yielded two digestion products of the expected sizes (Fig. 1F). We also used our CRISPR/Cas9 system to disrupt the endogenous MR1 genes of the THP-1 and K562 cell lines. Transient transfection of K562 and THP-1 cells with CRISPR/Cas9 plasmid DNA did not allow MR1 gene editing with high enough frequencies for monitoring with the Surveyor assay. However, CRISPR/Cas9 lentivirus transduction successfully generated MR1 indels in both cell lines (Fig. 1G, 1H). In the case of THP-1, we used an enrichment strategy based on the positive selection of MR1 mutants able to escape killing by a MAIT cell clone. Following 7 d of coculture with MAIT cells at an E:T ratio of 1:2, we observed an increase in the frequency of mutations in the MR1 gene within the expanding THP-1 population, suggesting that the process allowed for the selection of disruptive MR1 mutations (Fig. 1H).

**Table I.** CRISPR/Cas9 sgRNA target sequences within the MR1 gene tested in this study

gRNA	Target Sequence (5′→3′)	PAM (5′→3′)	Exon	Strand
A	GGATGGGATCCGAAACGCCC	AGG	2	+
B	GGTGAAGCACAGCGATTCC	CGG	1	−
C	GTCCCTGAATTTATTTCGGT	TGG	2	−
D	GAACCTCGCGCCTGATCACT	GGG	2	−
E	GCAGTATGCATATGACGGGC	AGG	3	−

Target sequences were identified by searching for GN_19_GG DNA motifs on either strand of the MR1 cDNA sequence. We selected five nonoverlapping target sequences located in the first half of the MR1 coding sequence.

### Generation of MR1^−/−^ A549 clones: phenotypic and genotypic characterization

**FIGURE 2.**
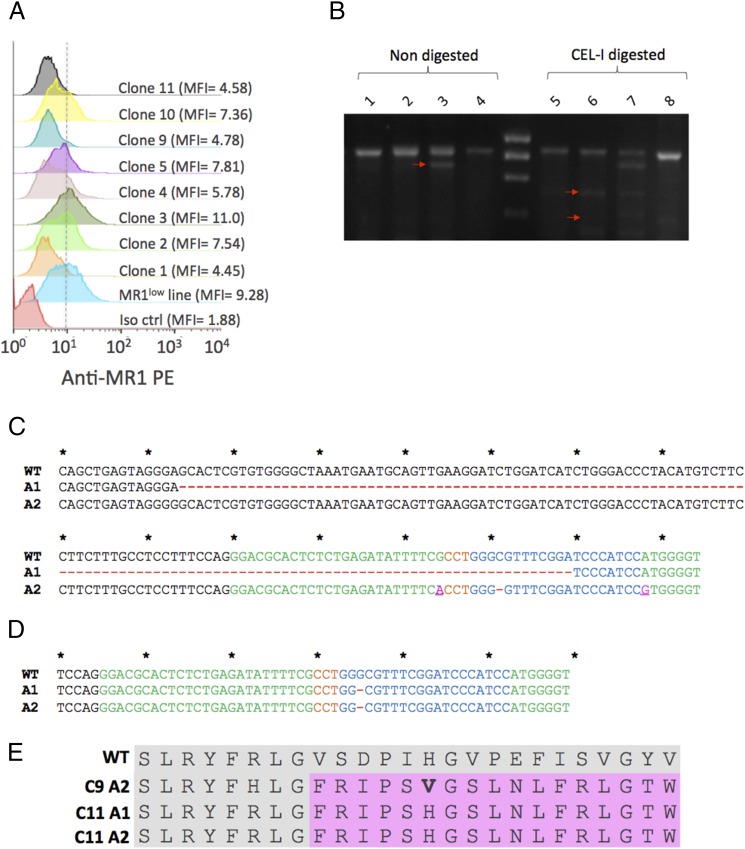
Characterization of A549 clonal derivatives bearing biallelic disruptive mutations in the MR1 gene. (**A**) Flow cytometry analysis of MR1 expression in eight different A549 clones obtained by limiting dilution and comparison with the bulk-transfected A549 parental cell line. Cells were treated overnight with acetyl-6-formylpterin (50 μg/ml) prior to staining with a PE-conjugated anti-MR1 Ab and data acquisition. The histogram plot shown is representative of three experiments. (**B**) Molecular characterization of mutations at the MR1 locus target site. PCR amplicons of WT A549 cells (*lane 1*) and A549 clones 11 (*lane 2*), 9 (*lane 3*), and 1 (*lane 4*) are shown in the absence of hybridization and CEL-I digestion. The Surveyor assay was performed with a total of 500 ng PCR amplicon obtained from genomic DNA of unmodified A549 cells or from the three different clones. Homoduplex WT DNA (*lane 5*) or heteroduplexes of annealed WT and modified amplicons from clone 11 (*lane 6*), clone 9 (*lane 7*), or clone 1 (*lane 8*) were digested with the CEL-I DNA-mismatch–specific enzyme. The gel shown is representative of at least four independent experiments. (**C**) Analysis of the MR1 genomic sequences recovered from A549 clone 9. The reference WT sequence is shown on top and the sequences recovered from clone corresponding to each allele are shown underneath. (**D**) WT MR1 reference sequence (*top*) and sequences corresponding to each allele of A549 clone 11 are shown. Nucleotide deletions are shown as red hyphens, and substitutions are shown as purple characters. Nucleotides corresponding to the sequence of the exon 2 MR1 are shown as green characters, the 19-nt gRNA.A target sequence is shown in blue characters, and the PAM sequence is shown in orange. (**E**) Predicted primary structures of the mutant MR1 proteins as determined by translating PCR amplicon DNA sequences. MR1 amino acids 26–50 are shown as alignments. The WT MR1 protein sequence is indicated as gray shadows and out-of-frame reads are shown as pink shadows. C9A2, clone 9 allele 2; C11A1, clone 11 allele 1; C11A2, clone 11 allele 2.

Although CRISPR/Cas9 gene disruption by lentiviral transduction appeared more efficient than transient expression, we reasoned that continuous expression of the gene-editing elements from a stable, integrated vector within the cells was more likely to generate detrimental off-target DNA cleavage, and we therefore elected to use bulk-transfected A549 cells to derive MR1^−/−^ clonal populations by limiting dilution. Following a first screen by flow cytometry of 16 clonal A549 derivatives (not shown), we selected 8 candidates showing homogeneous cell surface MR1 protein expression. The putative clones 1, 9, and 11 consistently displayed the lowest MFIs (Fig. 2A) and were taken forward for further molecular analysis of their genomic DNA. Interestingly, PCR amplification of the region flanking the sgRNA.A target sequence yielded two products in the case of clone 9 (Fig. 2B). In addition to a PCR amplicon of the predicted size (852 bp) common to all samples, a smaller fragment, shorter by 100–150 bp, was amplified, likely indicating a deletion near the site of Cas9 cleavage. Digestion of DNA heteroduplexes with the CEL-I enzyme generated additional bands matching digestion product sizes predicted by the positioning of the gRNA.A target and PAM sequences within the amplicon (∼520 and 330 bp) for clones 9 and 11 but not clone 1 (Fig. 2B). In the case of clone 9, further characterization of PCR products by Sanger sequencing revealed a 126-nt deletion spanning the intron/exon junction and resulting in the partial deletion of the CRISPR/Cas9 target sequence on one allele (Fig. 2C). The other allele bore a single nucleotide deletion as well as two base substitutions compared with the MR1 reference sequence (Fig. 2C), in line with the PCR analysis. Although it is unclear what the exact consequence the large deletion has on MR1 protein structure, the latter mutation likely disrupts the protein reading frame from amino acid 34 onward (Fig. 2E). In the case of clone 11, an identical single nucleotide deletion within the sgRNA target sequence could be detected on each allele (Fig. 2D). The reading frame on both alleles of clone 11’s coding sequence therefore shows the same frame shift as allele 2 of clone 9, where the native MR1 protein sequence is disrupted from amino acid 34 onward (Fig. 2E). Collectively, the lack of MR1 staining by flow cytometry and the presence of disruptive mutations on each allele for both clones indicated that MR1 expression was almost certainly abrogated as a result of CRISPR/Cas9 mutagenesis.

### MR1 CRISPR/Cas9 mutagenesis resulted in an unintended nucleotide deletion in the RP11-46A10.6 pseudogene

**FIGURE 3.**
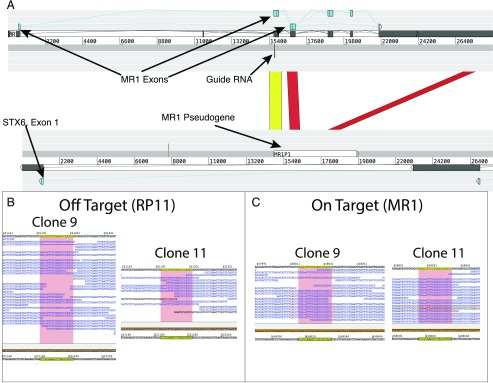
Results of whole-genome sequencing to identify off-target effects. (**A**) Figure generated from the Artemis comparison tool comparing the DNA sequence of MR1 to the RP11-46A10.6 pseudogene (located between exon 1 and 2 of STX6 gene). The red ribbons indicate large contiguous regions of 90%+ homology between the two genes, with the yellow ribbon being the region that contains the sequence targeted by the gRNA. The exons of MR1, STX6, and the RP11-46A10.6 pseudogene are annotated in this region and are shown in turquoise, dark gray, and white, respectively. (**B**) Unintended on-target effects within RP11-46A10.6 in samples clone 9 and clone 11. Two potential effects appear to be evident: one single base deletion seen in both clone 9 and clone 11, and one longer deletion in clone 9 (third read down from the top). The gRNA sequence is highlighted in yellow, and SNPs relative to the reference are shown in red characters. (**C**) On-target effects on MR1 in samples clone 9 and clone 11. The single base deletion observed in both clones, which is identical to that in RP11-46A10.6 relative to the gRNA target sequence, is evident for the vast majority of reads. The gRNA sequence is highlighted in yellow, and single nucleotide polymorphisms relative to the reference are shown in red characters.

To establish whether there were off-target effects resulting from the CRISPR/Cas9 modification, we undertook whole-genome sequencing for A549 clones 9 and 11. We identified likely off-target sites that occurred within genes (defined as sites varying at up to five bases compared with the MR1gRNA target sequence), totaling six possible genes where off-target effects could have been observed. Mapping the whole-genome sequence data back to the reference sequences for these genes, and for MR1, we identified only one case of unintended mutation. This was in a pseudogene bearing a high degree of sequence homology (94%) with the targeted MR1 region (Fig. 3A) and containing the exact same gRNA target sequence (Fig. 3B). In both samples the same nucleotide deletion at position 16 of the gRNA sequence was observed at this locus as on both clone 11 MR1 loci and on one MR1 locus of clone 9 (Figs. 2C, 2D, 3C). Another coding gene (STX6) sits within the RP11-46A10.6 region. However, the exons making up the STX6 protein–coding sequence are a considerable distance from the RP11-46A10.6 target sequence (Fig. 3A). We also examined our sequence reads to identify any other off-target effects that had longer deletions. Other than the one identified in MR1 for clone 9, we observed no evidence for other long deletions in the reads generated as part of the whole-genome sequencing (not shown).

### A549 mutant clones infected with bacteria selectively fail to activate MAIT cell clones

**FIGURE 4.**
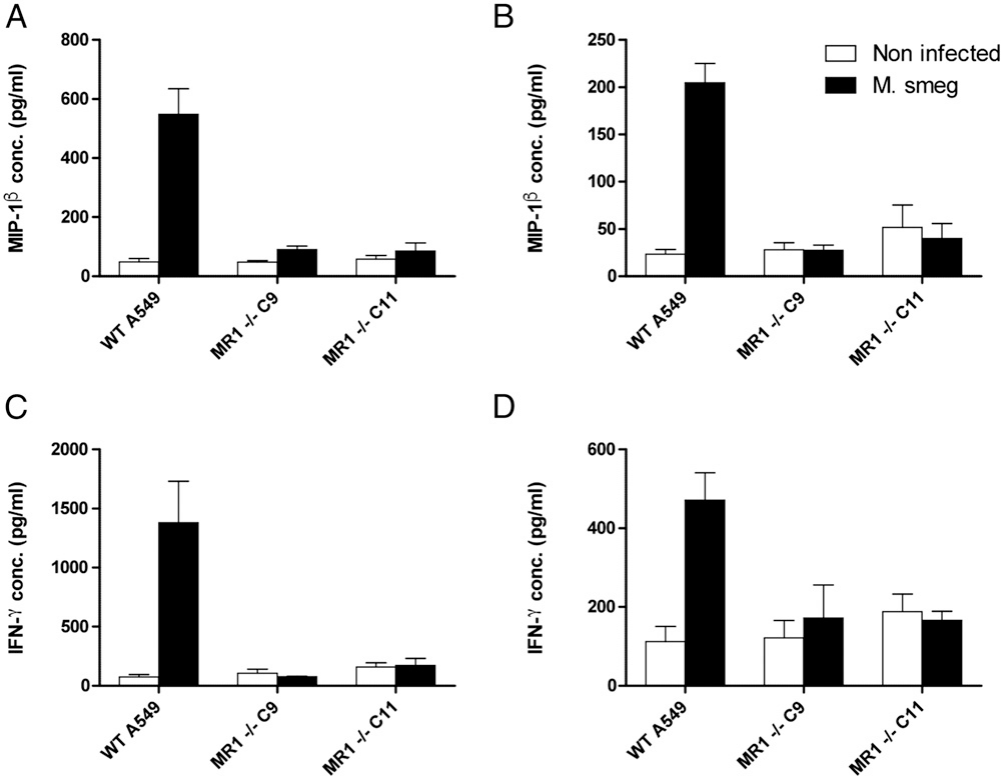
MAIT cells fail to recognize A549 derivatives clone 9 and clone 11 infected with *M. smegmatis*. The D481A9 and 426B1 MAIT cell clones were cocultured with the indicated A549 APCs infected with *M. smegmatis* or not as described in *Materials and Methods*. Supernatants were collected following overnight incubation. MIP-1β produced by MAIT cell clones D481A9 (**A**) and D426B1 (**B**), respectively, were quantified by ELISA. IFN-γ levels were also measured for both D481A9 (**C**) and D426B1 (**D**). Assays were carried out in triplicate wells. Means ± SEM are shown on the graph using data representative of three experiments.

Next we sought to confirm MR1 deficiency at the functional level. WT A549 cells or clones 9 and 11 infected with *M. smegmatis* were cocultured with the MAIT cell clones D426B1 and D481A9 known to express canonical TRAV1-2_TRAJ33 MAIT TCR α-chain rearrangement differing in their CDR3 sequences and two distinct β-chains (TRBV6-4 for D426B1 and TRBV20-1 for D481A9) (2, 12). In contrast to infected WT A549s, which triggered efficient MIP-1β and IFN-γ release, MR1-deficient clones 9 and 11 failed to activate either MAIT cell clone (Fig. 4). These results show that both A549 clonal derivatives are unable to present bacterial Ags in the context of cell-surface MR1. Such a distinct phenotype implies that CRISPR/Cas9 mutagenesis induced biallelic disruptive mutations, resulting in loss of MR1 protein function in both A549 clones.

### MR1 mutations do not affect the expression of nonclassical or classical HLA molecules

**FIGURE 5.**
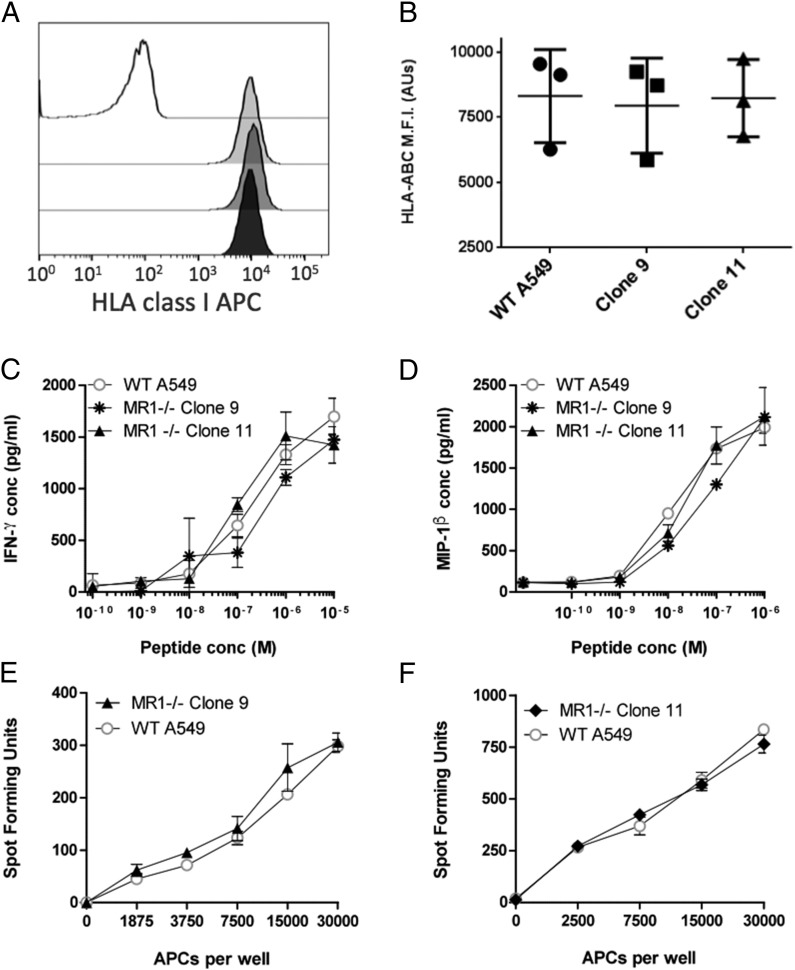
MR1 disruption does not affect HLA class Ia and HLA-E Ag presentation. (**A**) Representative cell surface HLA-I expression by A549 cells. MFI values were 56.4 (isotype control), 8733 (WT A549), 9880 (clone 11), and 8682 (clone 9). (**B**) MFI from three surface HLA-I staining of A549 WT and MR1^−/−^ cells. Individual MFI datum points as well as mean values ± SEM are shown. The data shown are representative of two independent staining experiments. (**C** and **D**) Secretion of IFN-γ and MIP-1β cytokines by CD8^+^ T cell clones B9 in response to increasing concentrations of an agonist peptide (SELEIKRY) derived from the EBV BZLF1 protein and presented by HLA-B*1801 expressed on the surface of WT A549, clone 9, or clone 11 as indicated in the key. (**E**) Activation of the D160 1–23 HLA-E–restricted CD8^+^ T cell clone in response to increasing numbers of WT A549 or A549 MR1^−/−^ clone 9 cells exposed to pronase-digested *M. tuberculosis* cell wall extracts. (**F**) Activation of the D160 1–23 HLA-E–restricted CD8^+^ T cell clone measured as spot-forming units in response to increasing numbers of WT A549 or A549 MR1^−/−^ clone 11 exposed to pronase-digested *M. tuberculosis* cell wall extracts in ELISPOT assays. The data shown are representative of two independent experiments.

To further assess the genetic integrity of both mutant A549 clones, we performed additional phenotypic and functional experiments. These established that cell-surface expression levels of classical HLA-I molecules were not affected in MR1-deficient cells (Fig. 5A, 5B). Moreover, both MR1^−/−^ A549 clones were able to present HLA-B*1801–restricted peptides derived from the EBV protein BZLF1 to a CD8^+^ T cell clone with efficiencies similar to the WT A549 cells, as evidenced by equivalent cytokine release in peptide titration experiments (Fig. 5C, 5D). Finally, when cocultured with the HLA-E–restricted CD8^+^ T cell clone D160 1–23, the A549 clones 9 and 11 presenting *M. tuberculosis* cell wall fractions triggered similar IFN-γ secretion levels compared with the WT A549 cells (Fig. 5E, 5F). Collectively, these data suggest that classical and nonclassical HLA-I Ag presentation is intact in the MR1-deficient cells.

### Generation and functional characterization of MR1^−/−^ THP-1 clones

**FIGURE 6.**
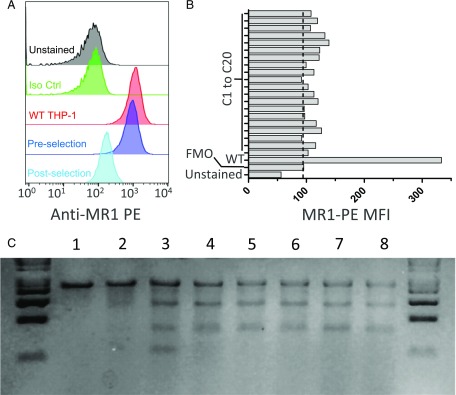
Isolation and characterization of THP-1 clonal derivatives bearing disruptive mutations in the MR1 gene. (**A**) Flow cytometry analysis of MR1 expression in THP-1 cells transduced with MR1 CRISPR/Cas9 lentiviral particles before and after enrichment of MR1-deficient cells by selection with the 426B1 MAIT cell clone, as indicated in the key to the figure. MFI values were: unstained, 45.5; isotype control, 53.9; WT THP-1, 1033; preselection edited cells, 820; and postselection edited cells, 161. (**B**) Staining of 20 different THP-1 clones obtained by limiting dilution from the postselection THP-1 parental cell line. MFI values of the indicated populations are represented. The data shown are representative of two independent experiments. (**C**) Molecular characterization of mutations at the MR1 locus target site. CEL-I–digested PCR amplicon homoduplexes of WT THP-1 cells (*lane 1*), heteroduplexes of WT THP-1 cells DNA hybridized with DNA from bulk-transduced THP-1 preselection (*lane 2*) and postselection (*lane 3*), as well as clones 3 (*lane 4*), 6 (*lane 5*), 7 (*lane 6*), 11 (*lane 7*), and 13 (*lane 8*). The Surveyor assay was performed with a total of 500 ng PCR amplicon obtained from genomic DNA of unmodified THP-1 cells or from the different bulk and clonal populations. The gel shown is representative of two independent experiments.

**FIGURE 7.**
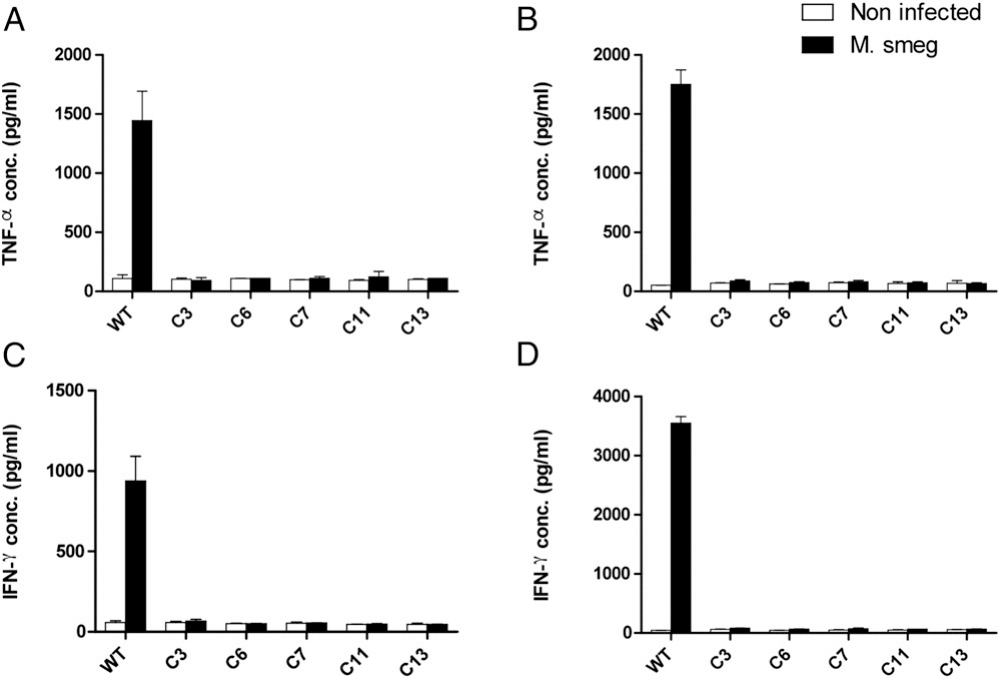
THP-1 single-cell derivatives infected with *M. smegmatis* are unable to activate MAIT cells. The D481A9 and 426B1 MAIT cell clones were cocultured with the indicated THP-1 derivatives infected with *M. smegmatis* or not as described in *Materials and Methods*. Supernatants were collected following overnight incubation. TNF-α produced by MAIT cell clones D481A9 (**A**) and D426B1 (**B**), respectively, was quantified by ELISA. IFN-γ levels were also measured for both D481 (**C**) and D426B1 (**D**). Assays were carried out in triplicate wells. Means ± SEM are shown on the graph using data representative of two experiments. Assays were carried out in triplicate wells.

The monocytic THP-1 cell line is a broadly used and well-characterized tool to study bacterial phagocytosis and Ag presentation (35). The availability of MR1^−/−^ THP-1 cells would likely be of interest to study MAIT cell responses to a range of pathogens. Following the selection of bulk-transduced THP-1 cells bearing increased MR1 mutations that conferred resistance to MAIT cell killing (Fig. 1H), we sought to derive clonal populations by single-cell dilution and assessed their MR1 phenotype. MR1 protein expression, as measured by flow cytometry, was markedly reduced in bulk THP-1 cells that underwent the positive selection process compared with the initial transduced preselection population (Fig. 6A), confirming the mismatch nuclease assay (Figs. 1H, 6C). All 20 tested THP-1 derivatives isolated by single-cell dilution of the selected population showed MR1 expression levels similar to those of the negative control used in the assay (Fig. 6B). Moreover, CEL-I digestion of five of these cell populations pointed to the presence of mismatch mutations at high frequencies within MR1 (Fig. 6C). Taken together, these data suggested that many, if not most, THP-1 derivatives bore biallelic disruptive MR1 mutations. Next we tested the ability of THP-1 cells to activate MAIT cells following *M. smegmatis* infection. Similar to the A549 clone 9 and clone 11, all the tested THP-1 derivatives failed to activate both D426B1 and D481A9 MAIT cell clones, as measured in cytokine release assays (Fig. 7), whereas the infected parental THP-1 cells induced the secretion of high levels of both TNF-α and IFN-γ by D481A9 (Fig. 7A, 7C) by D426B1 (Fig. 7B, 7D), thereby demonstrating that these cells are unable to present MR1-restricted Ags and confirming their knockout status.

## Discussion

RNA-guided genome engineering with CRISPR/Cas9 is a versatile and efficient tool to manipulate the genome of cell lines, including somatic and germinal cells. We have adapted this technology in a novel all-in-one lentiviral vector for the simultaneous delivery of the Cas9 endonuclease and an associated sgRNA. We designed sgRNA target sequences specific for the human MR1 locus and, following an initial screen in a reporter system, selected a target sequence we used to efficiently disrupt the expression of endogenous MR1 in three cell lines. We subsequently expanded clonal A549 and THP-1 populations bearing disruptive biallelic MR1 mutations. T cell assays where MAIT cells were activated by the parental A549 and THP-1 cell lines but failed to recognize cells obtained by limiting dilution upon infection with *M. smegmatis* confirmed MR1 protein loss of function. Importantly, CRISPR/Cas9-directed mutagenesis in these cells did not alter Ag processing and presentation by classical and nonclassical HLAs, highlighting the specificity of the genome editing approach and validating the use of these two clones to accurately characterize MR1-restricted Ags and their recognition by MAIT cells.

The two MR1-deficient A549 clones characterized in this study were generated from transient expression of an sgRNA and Cas9 elements in the parental cell line. The expression level of MR1 on the surface of A549 cells is relatively weak, and monitoring by flow cytometry, even after stabilization with high acetyl-6-formylpterine concentrations, did not allow us to unambiguously identify a population of MR1^−^ cells in the bulk-transfected population (Fig. 1E). However, the CEL-I mismatch-specific nuclease assay clearly showed that mutations were introduced at the intended site within the MR1 gene (Fig. 1F). The level of MR1 disruption was increased following flow cytometry cell sorting of an MR1^−/low^ population (not shown) from which we isolated and expanded individual clones by limiting dilution. A screen of 16 clonal populations identified eight derivatives that exhibited MR1 MFIs consistently lower than those of the parental cells (Fig. 2A). Three of these clones appeared MR1^−^ by flow cytometry and were taken forward for molecular analysis of genomic DNA. Disruptions at the MR1 locus could be identified in two clones using the CEL-I assay, and Sanger sequencing confirmed the presence of frameshift mutations on both alleles for each clone (Fig. 2C). Overall 2 of 16 of the clones (12.5%) displayed the genotype of interest. This suggested that extending the induction of disruptive MR1 mutations in other cell lines and, possibly, in primary cells using this system should be possible. We confirmed this by applying CRISPR/Cas9 mutagenesis to K562 and THP-1 cells. In both lines we were able to show the induction of MR1 mutations at the intended target site with the mismatch nuclease assay (Fig. 1G, 1H). In the case of the highly phagocytic THP-1 cell line, we set up a mutation enrichment strategy relying on the positive selection of THP-1 mutants unable to present MR1 Ags following infection with *M. smegmatis* and on MAIT cell cytotoxicity as a selecting force. The frequency of MR1 mutations was markedly increased by this selection process (Figs. 1H, 6A, 6C), and we were able to isolate monoclonal THP-1 cells by limiting dilution and expansion. All these derivatives failed to stain with anti-MR1 Ab (Fig. 6A, 6B), and the five we tested functionally did not activate MAIT cells in cytokine release assays (Fig. 7). Although we did not establish the clonal nature of these populations, it seems likely that at least some of them are MR1^−/−^ clones that can be used to study intracellular bacteria of different species than those infecting the A549 cells. The fact that all the THP-1 derivatives failed to activate MAIT cells (Fig. 7) is a clear indication of the efficiency of the positive selection approach we undertook, which could prove a useful and powerful way to establish the MHC restriction or Ag specificity of T cells without having to generate clonal target cell derivatives bearing biallelic mutations. Single-cell cloning is a cumbersome process and can be impossible to implement in the context of primary cells with limited proliferative capacity. Moreover, it is conceivable that the efficiency of this approach can be accurately quantified through a deep amplicon sequencing approach.

Concerning the nature of the A549 genomic mutations, it is notable that, on one allele of clone 9, we identified a 126-bp deletion spanning the junction between MR1 exon 2 and the flanking upstream intronic region and ending at the sgRNA target sequence (Fig. 2C). Such large deletions are not commonly observed following nonhomologous end joining genome editing with nuclease systems (CRISPR/Cas9 or others). The genomic DNA disruptions observed on the three other alleles were more in line with what is described in the literature, as we identified a single nucleotide deletion common to all three other alleles that disrupted the protein reading frame from amino acid 34 onward (Fig. 2C–E).

Although the MR1 target sequence we used bears little homology with other classical and nonclassical HLA genes, it was important to test whether other Ag presentation pathways had been disrupted as a result of the genome editing process. Albeit not exhaustive, our control experiments showed that classical HLA class I expression (Fig. 5A, 5B) as well as presentation of cognate exogenous peptide to an HLA-B*1801–restricted CD8^+^ T cell clone (Fig. 5C, 5D) by both A549 derivatives were equivalent to the unmodified A549 cells. Moreover, both clones activated an HLA-E–restricted CD8^+^ T cell clone with efficiencies similar to WT A549 (Fig. 5E, 5F). Collectively, these results imply that both the expression and processing of HLA alleles as well as peptide presentation are intact in these cells. Additionally, the basic properties of A549 clones 9 and 11 such as growth rate and morphology appeared unaffected (not shown); however, this does not exclude the possibility of other off-target mutations in unrelated genes. To address this possibility, we performed whole-genome sequencing on both A549 clones and searched for mutations in genes containing sequences with homology to our MR1 gRNA target. In total we examined 7580 locations with off-target potential across the genome. We found no mutations in these except in the RP11-46A10.6 pseudogene (Fig. 3B) locus that contains DNA regions of high homology with some MR1 introns and exons (Fig. 3A). Most notably, the 23 nucleotides composing the MR1 gRNA target sequence and adjacent PAM are 100% conserved in RP11-46A10.6 (Fig. 3B, 3C). Interestingly, the RP11-46A10.6 A549 loci contained the same mutation we identified as being the most common in MR1, that is, a single base deletion at position 17 of the sgRNA seed region. Even though this mutation was clearly unintended, it is on-target in the sense that it occurred at a locus bearing the full intact sgRNA target and PAM sequences. The potential for such an unintended effect was missed during the initial assessment of our MR1 gRNA candidates because we only searched for gRNA target and PAM sequence homology against coding genomic regions using the BLAST tool. Both A549 clones bear this pseudogene mutation, most likely in a homozygous manner, yet we anticipate no functional consequences given the noncoding nature of this locus. The fact that our sgRNA induced mutation in both MR1 and the RP11-46A10.6 pseudogene, where there is a 100% conservation of sequence, serves to highlight both the high efficiency and on-target specificity of CRISPR/Cas9.

The availability of MR1-deficient cells should be useful for fundamental investigations into the biology of MR1 and MR1-restricted T cells as well as for the potential development of therapeutic and diagnostic tools seeking to harness this invariant nonclassical HLA molecule. First, MR1^−/−^ cells will help with the unambiguous identification of MR1-restricted T cells because, unlike Ab blockade, the absence of MR1 completely obliterates MAIT cell responses to bacterially infected cells (Fig. 4). Second, in in vitro activation experiments, MR1-deficient APCs should allow characterizing and quantifying MR1-restricted T cells within the bulk pathogen-specific T cell response. Third, it is unclear at present whether bacterial Ags presented by MR1 show a degree of chemical and structural variability (36) and whether responding T cells can discriminate between distinct cognate MR1/Ag complexes via their clonal TCR. MR1^−/−^ cells should be a useful tool to test the ability of individual putative MR1 ligands to activate T cells. This would require obtaining chemically pure compounds used to pulse MR1-sufficient or -deficient APCs to test the response of T cell clones with distinct TCR usage and assess MR1 restriction. Fourth, the availability of cell lines in which endogenous MR1 expression is abrogated could also prove useful to knock-in mutated or chimeric reporter MR1 proteins and characterize the cellular biology of MR1, such as its trafficking and distribution in subcellular compartments. Such an approach may help shed light on the mechanisms governing the processing and loading of vitamin B derivatives without interference from endogenous MR1 molecules. Finally, MR1^−/−^ cells are likely to prove valuable for the generation and/or validation of biologicals and compounds targeting MR1.

In summary, we have generated THP-1 and A549 isogenic cells deficient for MR1 and a CRISPR/Cas9 lentivector that should allow applying the same process to other cell types. These reagents should facilitate investigations seeking to further understand the mechanisms underpinning the unique biology of MR1 and to harness its therapeutic and diagnostic potential. In the future, similar genome editing approaches could also be extended to facilitate basic and applied research on other invariant MHC-related proteins such as the CD1 family, HLA-E, or other MHC class Ib gene products that may serve as ligands for the TCR (37).

## Supplementary Material

Data Supplement
