# Supplementary material for: Engineering of Isogenic Cells Deficient for MR1 with a CRISPR/Cas9 Lentiviral System: Tools To Study Microbial Antigen Processing and Presentation to Human MR1-Restricted T Cells
Source: J Immunol. 2016 Jun 15;197(3):971–82. doi: 10.4049/jimmunol.1501402 (PMC4947828; doi:10.4049/jimmunol.1501402)
Supplement: Data Supplement [file JI_1501402.zip › JI_1501402_Supplemental_Material_1.pdf]

gRNA target sequence PCR cloning primers	**gRNA_A_Fwd**	GAAACGCCC**GTTTTAGAGCTAGAAATAGCAAGTTAA**
	**gRNA_A_Rev**	GGATCCCATC**CGGTGTTTCGTCCTTTCC**
	**gRNA_B_Fwd**	CAGCGATTCC**GTTTTAGAGCTAGAAATAGCAAGTTAA**
	**gRNA_B_Rev**	TGCTTCAC**CGGTGTTTCGTCCTTTCC**
	**gRNA_C_Fwd**	AATTTATTTCGGT**GTTTTAGAGCTAGAAATAGCAAGTTAA**
	**gRNA_C_Rev**	CAGGGA**CGGTGTTTCGTCCTTTCC**
	**gRNA_D_Fwd**	GCCTGATCACT**GTTTTAGAGCTAGAAATAGCAAGTTAA**
	**gRNA_D_Rev**	GCGAGGTT**CGGTGTTTCGTCCTTTCC**
	**gRNA_E_Fwd**	TATGACGGGC**GTTTTAGAGCTAGAAATAGCAAGTTAA**
	**gRNA_E_Rev**	TGCATACTG**CGGTGTTTCGTCCTTTCC**
Primers for genomic MR1 PCR and SURVEYOR assay	**SURV1_Fwd**	GCATGTGTTTGTGTGCCTGT
	**SURV1_Rev**	GGTGCAATTCAGCATCCGC
Primers for MR1 cDNA PCR and SURVEYOR assay	**SURV2_Fwd**	GGTCTTACTGACATCCACTTTGC
	**SURV2_Rev**	CAGTGATCAGGCGCGAG
MR1 amplicon cloning primers	**SURV_Fwd_BsaI**	gcgcGGTCTCcGCATGTGTTTGTGTGCCTGT
	**SURV_Rev_BsaI**	gcgcGGTCTCcTGCCGGTGCAATTCAGCATCCGC
Primers used to sequence MR1 amplicons	**Seq_Fwd**	CCAGTTGCTGAAGATCGCGAAGC
	**Seq_Rev**	TGCCACTCGATGTGATGTCCTC
Colony PCR primers flanking gRNA target sites	**pLKO.1-A**	GACTATCATATGCTTACCGT
	**gRNAcolPCR_R**	CACTTGATGTACTGCCAAGT
gRNA/pCMV-Cas9 PCR amplification in pCDNA.3	**pCDNA.3_Fwd**	GCACCGGTTGTACAAAAAAGCAGGCTTTA
	**pCDNA.3_Rev**	GCATGCATTCACACCTTCCTCTTCTTCT
"Empty" pRRL vector PCR amplification	**pRRL.0_Fwd**	GCATGCATAATCAACCTCTGGATTACAAAATTTG
	**pRRL.0_Rev**	ACCGGTGCTAGTCTCGTGATCGATAAAAT

Supplementary Table 1. List of primers used in project.

Thu Jan 22, 2015 23:17 -0000
pRRL_Cas9_MR1gRNA_A.str
Text Map

```
                                                                                                      100
                *         *         *         *         *         *         *         *         *         *
   1 AGCTTAATGTAGTCTTATGCAATACTCTTGTAGTCTTGCAACATGGTAACGATGAGTTAGCAACATGCCTTACAAGGAGAGAAAAAGCACCGTGCATGCC 100

                                                                                                      200
                *         *         *         *         *         *         *         *         *         *
 101 GATTGGTGGAAGTAAGGTGGTACGATCGTGCCTTATTAGGAAGGCAACAGACGGGTCTGACATGGATTGGACGAACCACTGAATTGCCGCATTGCAGAGA 200

                                                                                                      300
                *         *         *         *         *         *         *         *         *         *
 201 TATTGTATTTAAGTGCCTAGCTCGATACAATAAACGGGTCTCTCTGGTTAGACCAGATCTGAGCCTGGGAGCTCTCTGGCTAACTAGGGAACCCACTGCT 300
                                         >>>>>>
                                         BsaI site
                                        >>>>>>>>>>>>>>>>>>>>>>>>>>>>>>>>>>>>>>>>>>>>>>>>>>>>>>>>>>>>>>>>>
                                        HIV-1 5'LTR

                                                                                                      400
                *         *         *         *         *         *         *         *         *         *
 301 TAAGCCTCAATAAAGCTTGCCTTGAGTGCTTCAAGTAGTGTGTGCCCGTCTGTTGTGTGACTCTGGTAACTAGAGATCCCTCAGACCCTTTTAGTCAGTG 400
     >>>>>>>>>>>>>>>>>>>>>>>>>>>>>>>>>>>>>>>>>>>>>>>>>>>>>>>>>>>>>>>>>>>>>>>>>>>>>>>>>>>>>>>>>>>>>>>>>>>>
     HIV-1 5'LTR

                                                                                                      500
                *         *         *         *         *         *         *         *         *         *
 401 TGGAAAATCTCTAGCAGTGGCGCCCGAACAGGGACCTGAAAGCGAAAGGGAAACCAGAGCTCTCTCGACGCAGGACTCGGCTTGCTGAAGCGCGCACGGC 500
     >>>>>>>>>>>>>>>
     HIV-1 5'LTR

                                                                                                      600
                *         *         *         *         *         *         *         *         *         *
 501 AAGAGGCGAGGGGCGGCGACTGGTGAGTACGCCAAAAATTTTGACTAGCGGAGGCTAGAAGGAGAGAGATGGGTGCGAGAGCGTCAGTATTAAGCGGGGG 600
                            >>>>>>>>>>>>>>>>>>>>>>>>>>>>>>>>>>>>>>>>>>>>>
                            HIV-1_psI_pack

                                                                                                      700
                *         *         *         *         *         *         *         *         *         *
 601 AGAATTAGATCGCGATGGGAAAAAATTCGGTTAAGGCCAGGGGGAAAGAAAAAATATAAATTAAAACATATAGTATGGGCAAGCAGGGAGCTAGAACGAT 700

                                                                                                      800
                *         *         *         *         *         *         *         *         *         *
 701 TCGCAGTTAATCCTGGCCTGTTAGAAACATCAGAAGGCTGTAGACAAATACTGGGACAGCTACAACCATCCCTTCAGACAGGATCAGAAGAACTTAGATC 800

                                                                                                      900
                *         *         *         *         *         *         *         *         *         *
 801 ATTATATAATACAGTAGCAACCCTCTATTGTGTGCATCAAAGGATAGAGATAAAAGACACCAAGGAAGCTTTAGACAAGATAGAGGAAGAGCAAAACAAA 900

                                                                                                     1000
                *         *         *         *         *         *         *         *         *         *
 901 AGTAAGACCACCGCACAGCAAGCGGCCGCTGATCTTCAGACCTGGAGGAGGAGATATGAGGGACAATTGGAGAAGTGAATTATATAAATATAAAGTAGTA 1000

                                                                                                     1100
                *         *         *         *         *         *         *         *         *         *
1001 AAAATTGAACCATTAGGAGTAGCACCCACCAAGGCAAAGAGAAGAGTGGTGCAGAGAGAAAAAAGAGCAGTGGGAATAGGAGCTTTGTTCCTTGGGTTCT 1100
                                                                                  >>>>>>>>>>>>>>>>>>>>>>>
                                                                                  Rev response element

                                                                                                     1200
                *         *         *         *         *         *         *         *         *         *
1101 TGGGAGCAGCAGGAAGCACTATGGGCGCAGCCTCAATGACGCTGACGGTACAGGCCAGACAATTATTGTCTGGTATAGTGCAGCAGCAGAACAATTTGCT 1200
     >>>>>>>>>>>>>>>>>>>>>>>>>>>>>>>>>>>>>>>>>>>>>>>>>>>>>>>>>>>>>>>>>>>>>>>>>>>>>>>>>>>>>>>>>>>>>>>>>>>>
     Rev response element

                                                                                                     1300
                *         *         *         *         *         *         *         *         *         *
1201 GAGGGCTATTGAGGCGCAACAGCATCTGTTGCAACTCACAGTCTGGGGCATCAAGCAGCTCCAGGCAAGAATCCTGGCTGTGGAAAGATACCTAAAGGAT 1300
     >>>>>>>>>>>>>>>>>>>>>>>>>>>>>>>>>>>>>>>>>>>>>>>>>>>>>>>>>>>>>>>>>>>>>>>>>>>>>>>>>>>>>>>>>>>>>>>>>>>>
     Rev response element

                                                                                                     1400
```

```
          *         *         *         *         *         *         *         *         *         *
1301 CAACAGCTCCTGGGGATTTGGGGTTGCTCTGGAAAACTCATTTGCACCACTGCTGTGCCTTGGAATGCTAGTTGGAGTAATAAATCTCTGGAACAGATTT 1400
     >>>>>>>>>>>
     Rev response element

                                                                                                     1500
          *         *         *         *         *         *         *         *         *         *
1401 GGAATCACACGACCTGGATGGAGTGGGACAGAGAAATTAACAATTACACAAGCTTAATACACTCCTTAATTGAAGAATCGCAAAACCAGCAAGAAAAGAA 1500

                                                                                                     1600
          *         *         *         *         *         *         *         *         *         *
1501 TGAACAAGAATTATTGGAATTAGATAAATGGGCAAGTTTGTGGAATTGGTTTAACATAACAAATTGGCTGTGGTATATAAAATTATTCATAATGATAGTA 1600

                                                                                                     1700
          *         *         *         *         *         *         *         *         *         *
1601 GGAGGCTTGGTAGGTTTAAGAATAGTTTTTGCTGTACTTTCTATAGTGAATAGAGTTAGGCAGGGATATTCACCATTATCGTTTCAGACCCACCTCCCAA 1700

                                                                                                     1800
          *         *         *         *         *         *         *         *         *         *
1701 CCCCGAGGGGACCCGACAGGCCCGAAGGAATAGAAGAAGAAGGTGGAGAGAGAGACAGAGACAGATCCATTCGATTAGTGAACGGATCTCGACGGTATCG 1800

                                                                                                     1900
          *         *         *         *         *         *         *         *         *         *
1801 GTTAACTTTTAAAAGAAAAGGGGGGATTGGGGGGTACAGTGCAGGGGAAAGAATAGTAGACATAATAGCAACAGACATACAAACTAAAGAATTACAAAAA 1900
               >>>>>>>>>>>>>>>>
               cPPT

                                                                                                     2000
          *         *         *         *         *         *         *         *         *         *
1901 CAAATTACAAAAATTCAAAATTTTATCGATCACGAGACTAGCACCGGTGCCCGGTTGTACAAAAAAGCAGGCTTTAAAGGAACCAATTCAGTCGACTGGA 2000

                                                                                                     2100
          *         *         *         *         *         *         *         *         *         *
2001 TCCGGTACCAAGGTCGGGCAGGAAGAGGGCCTATTTCCCATGATTCCTTCATATTTGCATATACGATACAAGGCTGTTAGAGAGATAATTAGAATTAATT 2100
     >>>>>>>>>>>>>>>>>>>>>>>>>>>>>>>>>>>>>>>>>>>>>>>>>>>>>>>>>>>>>>>>>>>>>>>>>>>>>>>>>>>>>>>>>>>>>>>>>>>>
     U6 promoter

                                                                                                     2200
          *         *         *         *         *         *         *         *         *         *
2101 TGACTGTAAACACAAAGATATTAGTACAAAATACGTGACGTAGAAAGTAATAATTTCTTGGGTAGTTTGCAGTTTTAAAATTATGTTTTAAAATGGACTA 2200
     >>>>>>>>>>>>>>>>>>>>>>>>>>>>>>>>>>>>>>>>>>>>>>>>>>>>>>>>>>>>>>>>>>>>>>>>>>>>>>>>>>>>>>>>>>>>>>>>>>>>
     U6 promoter
                                                                                                >>>>>
                                                                                                pLKO.1A primer

                                                                                                     2300
          *         *         *         *         *         *         *         *         *         *
2201 TCATATGCTTACCGTAACTTGAAAGTATTTCGATTTCTTGGCTTTATATATCTTGTGGAAAGGACGAAACACCGGATGGGATCCGAAACGCCCGTTTTAG 2300
                                                                                              >>>>>>>
                                                                                              tracrRNA
     >>>>>>>>>>>>>>>>>>>>>>>>>>>>>>>>>>>>>>>>>>>>>>>>>>>>>>>>>>>>>>>>>>>>>>>>
     U6 promoter
     >>>>>>>>>>>>>>>                                                         >>>>>>>>>>>>>>>>>>>>
     pLKO.1A primer                                                          MR1 gRNA_A

                                                                                                     2400
          *         *         *         *         *         *         *         *         *         *
2301 AGCTAGAAATAGCAAGTTAAAATAAGGCTAGTCCGTTATCAACTTGAAAAAGTGGCACCGAGTCGGTGCTTTTTTTCTAGACCCAGCTTTCTTGTACAAA 2400
     >>>>>>>>>>>>>>>>>>>>>>>>>>>>>>>>>>>>>>>>>>>>>>>>>>>>>>>>>>>>>>>>>>>>>
     tracrRNA
                                                                         >>>>>>>
                                                                         RNApolIII termination

                                                                                                     2500
          *         *         *         *         *         *         *         *         *         *
2401 GTTGGCATTAACTAGTTATTAATAGTAATCAATTACGGGGTCATTAGTTCATAGCCCATATATGGAGTTCCGCGTTACATAACTTACGGTAAATGGCCCG 2500
                                                                            >>>>>>>>>>>>>>>>>>>>>>>>>>>>
                                                                            CMV promoter

                                                                                                     2600
          *         *         *         *         *         *         *         *         *         *
2501 CCTGGCTGACCGCCCAACGACCCCCGCCCATTGACGTCAATAATGACGTATGTTCCCATAGTAACGCCAATAGGGACTTTCCATTGACGTCAATGGGTGG 2600
```

```
     >>>>>>>>>>>>>>>>>>>>>>>>>>>>>>>>>>>>>>>>>>>>>>>>>>>>>>>>>>>>>>>>>>>>>>>>>>>>>>>>>>>>>>>>>>>>>>>>>>
     CMV promoter

                                                                                                      2700
              *         *         *         *         *         *         *         *         *         *
2601 AGTATTTACGGTAAACTGCCCACTTGGCAGTACATCAAGTGTATCATATGCCAAGTACGCCCCCTATTGACGTCAATGACGGTAAATGGCCCGCCTGGCA 2700
                         >>>>>>>>>>>>>>>>>>>>
                         gRNAcolPCR_R
     >>>>>>>>>>>>>>>>>>>>>>>>>>>>>>>>>>>>>>>>>>>>>>>>>>>>>>>>>>>>>>>>>>>>>>>>>>>>>>>>>>>>>>>>>>>>>>>>>>
     CMV promoter

                                                                                                      2800
              *         *         *         *         *         *         *         *         *         *
2701 TTATGCCCAGTACATGACCTTATGGGACTTTCCTACTTGGCAGTACATCTACGTATTAGTCATCGCTATTACCATGGTGATGCGGTTTTGGCAGTACATC 2800
     >>>>>>>>>>>>>>>>>>>>>>>>>>>>>>>>>>>>>>>>>>>>>>>>>>>>>>>>>>>>>>>>>>>>>>>>>>>>>>>>>>>>>>>>>>>>>>>>>>
     CMV promoter

                                                                                                      2900
              *         *         *         *         *         *         *         *         *         *
2801 AATGGGCGTGGATAGCGGTTTGACTCACGGGGATTTCCAAGTCTCCACCCCATTGACGTCAATGGGAGTTTGTTTTGGCACCAAAATCAACGGGACTTTC 2900
     >>>>>>>>>>>>>>>>>>>>>>>>>>>>>>>>>>>>>>>>>>>>>>>>>>>>>>>>>>>>>>>>>>>>>>>>>>>>>>>>>>>>>>>>>>>>>>>>>>
     CMV promoter

                                                                                                      3000
              *         *         *         *         *         *         *         *         *         *
2901 CAAAATGTCGTAACAACTCCGCCCCATTGACGCAAATGGGCGGTAGGCGTGTACGGTGGGAGGTCTATATAAGCAGAGCTCGTTTAGTGAACCGTCAGAT 3000
     >>>>>>>>>>>>>>>>>>>>>>>>>>>>>>>>>>>>>>>>>>>>>>>>>>>>>>>>>>>>>>>>>>>>>>>>>>>>>>>>>>>>>>>>>>>>>>>>>>
     CMV promoter

                                                                                                      3100
              *         *         *         *         *         *         *         *         *         *
3001 CGCCTGGAGACGCCATCCACGCTGTTTTGACCTCCATAGAAGACACCGGGACCGATCCAGCCTCCGGACTCTAGAGGATCGAACCCTTGCCACCATGGAC 3100
                                                                                              >>>>>>
                                                                                              Cas9 hscodop
           <<<<<<
           BsmBI site

                                                                                                      3200
              *         *         *         *         *         *         *         *         *         *
3101 AAGAAGTACTCCATTGGGCTCGATATCGGCACAAACAGCGTCGGCTGGGCCGTCATTACGGACGAGTACAAGGTGCCGAGCAAAAAATTCAAAGTTCTGG 3200
     >>>>>>>>>>>>>>>>>>>>>>>>>>>>>>>>>>>>>>>>>>>>>>>>>>>>>>>>>>>>>>>>>>>>>>>>>>>>>>>>>>>>>>>>>>>>>>>>>>
     Cas9 hscodop

                                                                                                      3300
              *         *         *         *         *         *         *         *         *         *
3201 GCAATACCGATCGCCACAGCATAAAGAAGAACCTCATTGGCGCCCTCCTGTTCGACTCCGGGGAGACGGCCGAAGCCACGCGGCTCAAAAGAACAGCACG 3300
     >>>>>>>>>>>>>>>>>>>>>>>>>>>>>>>>>>>>>>>>>>>>>>>>>>>>>>>>>>>>>>>>>>>>>>>>>>>>>>>>>>>>>>>>>>>>>>>>>>
     Cas9 hscodop
                                                             <<<<<<
                                                             BsmBI site

                                                                                                      3400
              *         *         *         *         *         *         *         *         *         *
3301 GCGCAGATATACCCGCAGAAAGAATCGGATCTGCTACCTGCAGGAGATCTTTAGTAATGAGATGGCTAAGGTGGATGACTCTTTCTTCCATAGGCTGGAG 3400
     >>>>>>>>>>>>>>>>>>>>>>>>>>>>>>>>>>>>>>>>>>>>>>>>>>>>>>>>>>>>>>>>>>>>>>>>>>>>>>>>>>>>>>>>>>>>>>>>>>
     Cas9 hscodop

                                                                                                      3500
              *         *         *         *         *         *         *         *         *         *
3401 GAGTCCTTTTTGGTGGAGGAGGATAAAAAGCACGAGCGCCACCCAATCTTTGGCAATATCGTGGACGAGGTGGCGTACCATGAAAAGTACCCAACCATAT 3500
     >>>>>>>>>>>>>>>>>>>>>>>>>>>>>>>>>>>>>>>>>>>>>>>>>>>>>>>>>>>>>>>>>>>>>>>>>>>>>>>>>>>>>>>>>>>>>>>>>>
     Cas9 hscodop

                                                                                                      3600
              *         *         *         *         *         *         *         *         *         *
3501 ATCATCTGAGGAAGAAGCTTGTAGACAGTACTGATAAGGCTGACTTGCGGTTGATCTATCTCGCGCTGGCGCATATGATCAAATTTCGGGGACACTTCCT 3600
     >>>>>>>>>>>>>>>>>>>>>>>>>>>>>>>>>>>>>>>>>>>>>>>>>>>>>>>>>>>>>>>>>>>>>>>>>>>>>>>>>>>>>>>>>>>>>>>>>>
     Cas9 hscodop

                                                                                                      3700
              *         *         *         *         *         *         *         *         *         *
3601 CATCGAGGGGGACCTGAACCCAGACAACAGCGATGTCGACAAACTCTTTATCCAACTGGTTCAGACTTACAATCAGCTTTTCGAAGAGAACCCGATCAAC 3700
```

```
     >>>>>>>>>>>>>>>>>>>>>>>>>>>>>>>>>>>>>>>>>>>>>>>>>>>>>>>>>>>>>>>>>>>>>>>>>>>>>>>>>>>>>>>>>>>>>>>>>
     Cas9 hscodop

                                                                                                 3800
                *         *         *         *         *         *         *         *         *         *
3701 GCATCCGGAGTTGACGCCAAAGCAATCCTGAGCGCTAGGCTGTCCAAATCCCGGCGGCTCGAAAACCTCATCGCACAGCTCCCTGGGGAGAAGAAGAACG 3800
     >>>>>>>>>>>>>>>>>>>>>>>>>>>>>>>>>>>>>>>>>>>>>>>>>>>>>>>>>>>>>>>>>>>>>>>>>>>>>>>>>>>>>>>>>>>>>>>>>
     Cas9 hscodop

                                                                                                 3900
                *         *         *         *         *         *         *         *         *         *
3801 GCCTGTTTGGTAATCTTATCGCCCTGTCACTCGGGCTGACCCCCAACTTTAAATCTAACTTCGACCTGGCCGAAGATGCCAAGCTTCAACTGAGCAAAGA 3900
     >>>>>>>>>>>>>>>>>>>>>>>>>>>>>>>>>>>>>>>>>>>>>>>>>>>>>>>>>>>>>>>>>>>>>>>>>>>>>>>>>>>>>>>>>>>>>>>>>
     Cas9 hscodop

                                                                                                 4000
                *         *         *         *         *         *         *         *         *         *
3901 CACCTACGATGATGATCTCGACAATCTGCTGGCCCAGATCGGCGACCAGTACGCAGACCTTTTTTTGGCGGCAAAGAACCTGTCAGACGCCATTCTGCTG 4000
     >>>>>>>>>>>>>>>>>>>>>>>>>>>>>>>>>>>>>>>>>>>>>>>>>>>>>>>>>>>>>>>>>>>>>>>>>>>>>>>>>>>>>>>>>>>>>>>>>
     Cas9 hscodop

                                                                                                 4100
                *         *         *         *         *         *         *         *         *         *
4001 AGTGATATTCTGCGAGTGAACACGGAGATCACCAAAGCTCCGCTGAGCGCTAGTATGATCAAGCGCTATGATGAGCACCACCAAGACTTGACTTTGCTGA 4100
     >>>>>>>>>>>>>>>>>>>>>>>>>>>>>>>>>>>>>>>>>>>>>>>>>>>>>>>>>>>>>>>>>>>>>>>>>>>>>>>>>>>>>>>>>>>>>>>>>
     Cas9 hscodop

                                                                                                 4200
                *         *         *         *         *         *         *         *         *         *
4101 AGGCCCTTGTCAGACAGCAACTGCCTGAGAAGTACAAGGAAATTTTCTTCGATCAGTCTAAAAATGGCTACGCCGGATACATTGACGGCGGAGCAAGCCA 4200
     >>>>>>>>>>>>>>>>>>>>>>>>>>>>>>>>>>>>>>>>>>>>>>>>>>>>>>>>>>>>>>>>>>>>>>>>>>>>>>>>>>>>>>>>>>>>>>>>>
     Cas9 hscodop

                                                                                                 4300
                *         *         *         *         *         *         *         *         *         *
4201 GGAGGAATTTTACAAATTTATTAAGCCCATCTTGGAAAAAATGGACGGCACCGAGGAGCTGCTGGTAAAGCTTAACAGAGAAGATCTGTTGCGCAAACAG 4300
     >>>>>>>>>>>>>>>>>>>>>>>>>>>>>>>>>>>>>>>>>>>>>>>>>>>>>>>>>>>>>>>>>>>>>>>>>>>>>>>>>>>>>>>>>>>>>>>>>
     Cas9 hscodop

                                                                                                 4400
                *         *         *         *         *         *         *         *         *         *
4301 CGCACTTTCGACAATGGAAGCATCCCCCACCAGATTCACCTGGGCGAACTGCACGCTATCCTCAGGCGGCAAGAGGATTTCTACCCCTTTTTGAAAGATA 4400
     >>>>>>>>>>>>>>>>>>>>>>>>>>>>>>>>>>>>>>>>>>>>>>>>>>>>>>>>>>>>>>>>>>>>>>>>>>>>>>>>>>>>>>>>>>>>>>>>>
     Cas9 hscodop

                                                                                                 4500
                *         *         *         *         *         *         *         *         *         *
4401 ACAGGGAAAAGATTGAGAAAATCCTCACATTTCGGATACCCTACTATGTAGGCCCCCTCGCCCGGGGAAATTCCAGATTCGCGTGGATGACTCGCAAATC 4500
     >>>>>>>>>>>>>>>>>>>>>>>>>>>>>>>>>>>>>>>>>>>>>>>>>>>>>>>>>>>>>>>>>>>>>>>>>>>>>>>>>>>>>>>>>>>>>>>>>
     Cas9 hscodop

                                                                                                 4600
                *         *         *         *         *         *         *         *         *         *
4501 AGAAGAGACCATCACTCCCTGGAACTTCGAGGAAGTCGTGGATAAGGGGGCCTCTGCCCAGTCCTTCATCGAAAGGATGACTAACTTTGATAAAAATCTG 4600
     >>>>>>>>>>>>>>>>>>>>>>>>>>>>>>>>>>>>>>>>>>>>>>>>>>>>>>>>>>>>>>>>>>>>>>>>>>>>>>>>>>>>>>>>>>>>>>>>>
     Cas9 hscodop
         <<<<<<
         BsaI site

                                                                                                 4700
                *         *         *         *         *         *         *         *         *         *
4601 CCTAACGAAAAGGTGCTTCCTAAACACTCTCTGCTGTACGAGTACTTCACAGTTTATAACGAGCTCACCAAGGTCAAATACGTCACAGAAGGGATGAGAA 4700
     >>>>>>>>>>>>>>>>>>>>>>>>>>>>>>>>>>>>>>>>>>>>>>>>>>>>>>>>>>>>>>>>>>>>>>>>>>>>>>>>>>>>>>>>>>>>>>>>>
     Cas9 hscodop

                                                                                                 4800
                *         *         *         *         *         *         *         *         *         *
4701 AGCCAGCATTCCTGTCTGGAGAGCAGAAGAAAGCTATCGTGGACCTCCTCTTCAAGACGAACCGGAAAGTTACCGTGAAACAGCTCAAAGAAGACTATTT 4800
     >>>>>>>>>>>>>>>>>>>>>>>>>>>>>>>>>>>>>>>>>>>>>>>>>>>>>>>>>>>>>>>>>>>>>>>>>>>>>>>>>>>>>>>>>>>>>>>>>
     Cas9 hscodop

                                                                                                 4900
```

```
4801 CAAAAAGATTGAATGTTTCGACTCTGTTGAAATCAGCGGAGTGGAGGATCGCTTCAACGCATCCCTGGGAACGTATCACGATCTCCTGAAAATCATTAAA 4900
     >>>>>>>>>>>>>>>>>>>>>>>>>>>>>>>>>>>>>>>>>>>>>>>>>>>>>>>>>>>>>>>>>>>>>>>>>>>>>>>>>>>>>>>>>>>>>>>>>
     Cas9 hscodop

                                                                                                   5000
4901 GACAAGGACTTCCTGGACAATGAGGAGAACGAGGACATTCTTGAGGACATTGTCCTCACCCTTACGTTGTTTGAAGATAGGGAGATGATTGAAGAACGCT 5000
     >>>>>>>>>>>>>>>>>>>>>>>>>>>>>>>>>>>>>>>>>>>>>>>>>>>>>>>>>>>>>>>>>>>>>>>>>>>>>>>>>>>>>>>>>>>>>>>>>
     Cas9 hscodop

                                                                                                   5100
5001 TGAAAACTTACGCTCATCTCTTCGACGACAAAGTCATGAAACAGCTCAAGAGGCGCCGATATACAGGATGGGGGCGGCTGTCAAGAAAACTGATCAATGG 5100
     >>>>>>>>>>>>>>>>>>>>>>>>>>>>>>>>>>>>>>>>>>>>>>>>>>>>>>>>>>>>>>>>>>>>>>>>>>>>>>>>>>>>>>>>>>>>>>>>>
     Cas9 hscodop

                                                                                                   5200
5101 GATCCGAGACAAGCAGAGTGGAAAGACAATCCTGGATTTTCTTAAGTCCGATGGATTTGCCAACCGGAACTTCATGCAGTTGATCCATGATGACTCTCTC 5200
     >>>>>>>>>>>>>>>>>>>>>>>>>>>>>>>>>>>>>>>>>>>>>>>>>>>>>>>>>>>>>>>>>>>>>>>>>>>>>>>>>>>>>>>>>>>>>>>>
     Cas9 hscodop

                                                                                                   5300
5201 ACCTTTAAGGAGGACATCCAGAAAGCACAAGTTTCTGGCCAGGGGGACAGTCTTCACGAGCACATCGCTAATCTTGCAGGTAGCCCAGCTATCAAAAAGG 5300
     >>>>>>>>>>>>>>>>>>>>>>>>>>>>>>>>>>>>>>>>>>>>>>>>>>>>>>>>>>>>>>>>>>>>>>>>>>>>>>>>>>>>>>>>>>>>>>>>
     Cas9 hscodop

                                                                                                   5400
5301 GAATACTGCAGACCGTTAAGGTCGTGGATGAACTCGTCAAAGTAATGGGAAGGCATAAGCCCGAGAATATCGTTATCGAGATGGCCCGAGAGAACCAAAC 5400
     >>>>>>>>>>>>>>>>>>>>>>>>>>>>>>>>>>>>>>>>>>>>>>>>>>>>>>>>>>>>>>>>>>>>>>>>>>>>>>>>>>>>>>>>>>>>>>>>
     Cas9 hscodop

                                                                                                   5500
5401 TACCCAGAAGGGACAGAAGAACAGTAGGGAAAGGATGAAGAGGATTGAAGAGGGTATAAAAGAACTGGGGTCCCAAATCCTTAAGGAACACCCAGTTGAA 5500
     >>>>>>>>>>>>>>>>>>>>>>>>>>>>>>>>>>>>>>>>>>>>>>>>>>>>>>>>>>>>>>>>>>>>>>>>>>>>>>>>>>>>>>>>>>>>>>>>
     Cas9 hscodop

                                                                                                   5600
5501 AACACCCAGCTTCAGAATGAGAAGCTCTACCTGTACTACCTGCAGAACGGCAGGGACATGTACGTGGATCAGGAACTGGACATCAATCGGCTCTCCGACT 5600
     >>>>>>>>>>>>>>>>>>>>>>>>>>>>>>>>>>>>>>>>>>>>>>>>>>>>>>>>>>>>>>>>>>>>>>>>>>>>>>>>>>>>>>>>>>>>>>>>
     Cas9 hscodop

                                                                                                   5700
5601 ACGACGTGGATCATATCGTGCCCCAGTCTTTTCTCAAAGATGATTCTATTGATAATAAAGTGTTGACAAGATCCGATAAAAATAGAGGGAAGAGTGATAA 5700
     >>>>>>>>>>>>>>>>>>>>>>>>>>>>>>>>>>>>>>>>>>>>>>>>>>>>>>>>>>>>>>>>>>>>>>>>>>>>>>>>>>>>>>>>>>>>>>>>
     Cas9 hscodop

                                                                                                   5800
5701 CGTCCCCTCAGAAGAAGTTGTCAAGAAAATGAAAAATTATTGGCGGCAGCTGCTGAACGCCAAACTGATCACACAACGGAAGTTCGATAATCTGACTAAG 5800
     >>>>>>>>>>>>>>>>>>>>>>>>>>>>>>>>>>>>>>>>>>>>>>>>>>>>>>>>>>>>>>>>>>>>>>>>>>>>>>>>>>>>>>>>>>>>>>>>
     Cas9 hscodop

                                                                                                   5900
5801 GCTGAACGAGGTGGCCTGTCTGAGTTGGATAAAGCCGGCTTCATCAAAAGGCAGCTTGTTGAGACACGCCAGATCACCAAGCACGTGGCCCAAATTCTCG 5900
     >>>>>>>>>>>>>>>>>>>>>>>>>>>>>>>>>>>>>>>>>>>>>>>>>>>>>>>>>>>>>>>>>>>>>>>>>>>>>>>>>>>>>>>>>>>>>>>>
     Cas9 hscodop

                                                                                                   6000
5901 ATTCACGCATGAACACCAAGTACGATGAAAATGACAAACTGATTCGAGAGGTGAAAGTTATTACTCTGAAGTCTAAGCTGGTCTCAGATTTCAGAAAGGA 6000
     >>>>>>>>>>>>>>>>>>>>>>>>>>>>>>>>>>>>>>>>>>>>>>>>>>>>>>>>>>>>>>>>>>>>>>>>>>>>>>>>>>>>>>>>>>>>>>>>
     Cas9 hscodop
                                                                                >>>>>>
                                                                                BsaI site
```

```
                                                                                                    6100
              *         *         *         *         *         *         *         *         *         *
6001 CTTTCAGTTTTATAAGGTGAGAGAGATCAACAATTACCACCATGCGCATGATGCCTACCTGAATGCAGTGGTAGGCACTGCACTTATCAAAAAATATCCC 6100
     >>>>>>>>>>>>>>>>>>>>>>>>>>>>>>>>>>>>>>>>>>>>>>>>>>>>>>>>>>>>>>>>>>>>>>>>>>>>>>>>>>>>>>>>>>>>>>>>>
     Cas9 hscodop

                                                                                                    6200
              *         *         *         *         *         *         *         *         *         *
6101 AAGCTTGAATCTGAATTTGTTTACGGAGACTATAAAGTGTACGATGTTAGGAAAATGATCGCAAAGTCTGAGCAGGAAATAGGCAAGGCCACCGCTAAGT 6200
     >>>>>>>>>>>>>>>>>>>>>>>>>>>>>>>>>>>>>>>>>>>>>>>>>>>>>>>>>>>>>>>>>>>>>>>>>>>>>>>>>>>>>>>>>>>>>>>>>
     Cas9 hscodop

                                                                                                    6300
              *         *         *         *         *         *         *         *         *         *
6201 ACTTCTTTTACAGCAATATTATGAATTTTTTCAAGACCGAGATTACACTGGCCAATGGAGAGATTCGGAAGCGACCACTTATCGAAACAAACGGAGAAAC 6300
     >>>>>>>>>>>>>>>>>>>>>>>>>>>>>>>>>>>>>>>>>>>>>>>>>>>>>>>>>>>>>>>>>>>>>>>>>>>>>>>>>>>>>>>>>>>>>>>>>
     Cas9 hscodop

                                                                                                    6400
              *         *         *         *         *         *         *         *         *         *
6301 AGGAGAAATCGTGTGGGACAAGGGTAGGGATTTCGCGACAGTCCGGAAGGTCCTGTCCATGCCGCAGGTGAACATCGTTAAAAAGACCGAAGTACAGACC 6400
     >>>>>>>>>>>>>>>>>>>>>>>>>>>>>>>>>>>>>>>>>>>>>>>>>>>>>>>>>>>>>>>>>>>>>>>>>>>>>>>>>>>>>>>>>>>>>>>>>
     Cas9 hscodop

                                                                                                    6500
              *         *         *         *         *         *         *         *         *         *
6401 GGAGGCTTCTCCAAGGAAAGTATCCTCCCGAAAAGGAACAGCGACAAGCTGATCGCACGCAAAAAAGATTGGGACCCCAAGAAATACGGCGGATTCGATT 6500
     >>>>>>>>>>>>>>>>>>>>>>>>>>>>>>>>>>>>>>>>>>>>>>>>>>>>>>>>>>>>>>>>>>>>>>>>>>>>>>>>>>>>>>>>>>>>>>>>>
     Cas9 hscodop

                                                                                                    6600
              *         *         *         *         *         *         *         *         *         *
6501 CTCCTACAGTCGCTTACAGTGTACTGGTTGTGGCCAAAGTGGAGAAAGGGAAGTCTAAAAAACTCAAAAGCGTCAAGGAACTGCTGGGCATCACAATCAT 6600
     >>>>>>>>>>>>>>>>>>>>>>>>>>>>>>>>>>>>>>>>>>>>>>>>>>>>>>>>>>>>>>>>>>>>>>>>>>>>>>>>>>>>>>>>>>>>>>>>>
     Cas9 hscodop

                                                                                                    6700
              *         *         *         *         *         *         *         *         *         *
6601 GGAGCGATCAAGCTTCGAAAAAAACCCCATCGACTTTCTCGAGGCGAAAGGATATAAAGAGGTCAAAAAAGACCTCATCATTAAGCTTCCCAAGTACTCT 6700
     >>>>>>>>>>>>>>>>>>>>>>>>>>>>>>>>>>>>>>>>>>>>>>>>>>>>>>>>>>>>>>>>>>>>>>>>>>>>>>>>>>>>>>>>>>>>>>>>>
     Cas9 hscodop

                                                                                                    6800
              *         *         *         *         *         *         *         *         *         *
6701 CTCTTTGAGCTTGAAAACGGCCGGAAACGAATGCTCGCTAGTGCGGGCGAGCTGCAGAAAGGTAACGAGCTGGCACTGCCCTCTAAATACGTTAATTTCT 6800
     >>>>>>>>>>>>>>>>>>>>>>>>>>>>>>>>>>>>>>>>>>>>>>>>>>>>>>>>>>>>>>>>>>>>>>>>>>>>>>>>>>>>>>>>>>>>>>>>>
     Cas9 hscodop

                                                                                                    6900
              *         *         *         *         *         *         *         *         *         *
6801 TGTATCTGGCCAGCCACTATGAAAAGCTCAAAGGGTCTCCCGAAGATAATGAGCAGAAGCAGCTGTTCGTGGAACAACACAAACACTACCTTGATGAGAT 6900
     >>>>>>>>>>>>>>>>>>>>>>>>>>>>>>>>>>>>>>>>>>>>>>>>>>>>>>>>>>>>>>>>>>>>>>>>>>>>>>>>>>>>>>>>>>>>>>>>>
     Cas9 hscodop
                                      >>>>>>
                                      BsaI site

                                                                                                    7000
              *         *         *         *         *         *         *         *         *         *
6901 CATCGAGCAAATAAGCGAATTCTCCAAAAGAGTGATCCTCGCCGACGCTAACCTCGATAAGGTGCTTTCTGCTTACAATAAGCACAGGGATAAGCCCATC 7000
     >>>>>>>>>>>>>>>>>>>>>>>>>>>>>>>>>>>>>>>>>>>>>>>>>>>>>>>>>>>>>>>>>>>>>>>>>>>>>>>>>>>>>>>>>>>>>>>>>
     Cas9 hscodop

                                                                                                    7100
              *         *         *         *         *         *         *         *         *         *
7001 AGGGAGCAGGCAGAAAACATTATCCACTTGTTTACTCTGACCAACTTGGGCGCGCCTGCAGCCTTCAAGTACTTCGACACCACCATAGACAGAAAGCGGT 7100
     >>>>>>>>>>>>>>>>>>>>>>>>>>>>>>>>>>>>>>>>>>>>>>>>>>>>>>>>>>>>>>>>>>>>>>>>>>>>>>>>>>>>>>>>>>>>>>>>>
     Cas9 hscodop

                                                                                                    7200
              *         *         *         *         *         *         *         *         *         *
7101 ACACCTCTACAAAGGAGGTCCTGGACGCCACACTGATTCATCAGTCAATTACGGGGCTCTATGAAACAAGAATCGACCTCTCTCAGCTCGGTGGAGACAG 7200
```

```
     >>>>>>>>>>>>>>>>>>>>>>>>>>>>>>>>>>>>>>>>>>>>>>>>>>>>>>>>>>>>>>>>>>>>>>>>>>>>>>>>>>>>>>>>>>>>>>>>>>>>
     Cas9 hscodop

7201 CAGGGCTGACCCCAAGAAGAAGAGGAAGGTGTGAATGCATAATCAACCTCTGGATTACAAAATTTGTGAAAGATTGACTGGTATTCTTAACTATGTTGCT 7300
     >>>>>>>>>>>>>>>>>>>>>>>>>>>>>>>>>>>        >>>>>>>>>>>>>>>>>>>>>>>>>>>>>>>>>>>>>>>>>>>>>>>>>>>>>>>>>>
     Cas9 hscodop                               WPRE

7301 CCTTTTACGCTATGTGGATACGCTGCTTTAATGCCTTTGTATCATGCTATTGCTTCCCGTATGGCTTTCATTTTCTCCTCCTTGTATAAATCCTGGTTGC 7400
     >>>>>>>>>>>>>>>>>>>>>>>>>>>>>>>>>>>>>>>>>>>>>>>>>>>>>>>>>>>>>>>>>>>>>>>>>>>>>>>>>>>>>>>>>>>>>>>>>>>>
     WPRE

7401 TGTCTCTTTATGAGGAGTTGTGGCCCGTTGTCAGGCAACGTGGCGTGGTGTGCACTGTGTTTGCTGACGCAACCCCCACTGGTTGGGGCATTGCCACCAC 7500
     >>>>>>>>>>>>>>>>>>>>>>>>>>>>>>>>>>>>>>>>>>>>>>>>>>>>>>>>>>>>>>>>>>>>>>>>>>>>>>>>>>>>>>>>>>>>>>>>>>>>
     WPRE

7501 CTGTCAGCTCCTTTCCGGGACTTTCGCTTTCCCCCTCCCTATTGCCACGGCGGAACTCATCGCCGCCTGCCTTGCCCGCTGCTGGACAGGGGCTCGGCTG 7600
     >>>>>>>>>>>>>>>>>>>>>>>>>>>>>>>>>>>>>>>>>>>>>>>>>>>>>>>>>>>>>>>>>>>>>>>>>>>>>>>>>>>>>>>>>>>>>>>>>>>>
     WPRE

7601 TTGGGCACTGACAATTCCGTGGTGTTGTCGGGGAAGCTGACGTCCTTTCCATGGCTGCTCGCCTGTGTTGCCACCTGGATTCTGCGCGGGACGTCCTTCT 7700
     >>>>>>>>>>>>>>>>>>>>>>>>>>>>>>>>>>>>>>>>>>>>>>>>>>>>>>>>>>>>>>>>>>>>>>>>>>>>>>>>>>>>>>>>>>>>>>>>>>>>
     WPRE

7701 GCTACGTCCCTTCGGCCCTCAATCCAGCGGACCTTCCTTCCCGCGGCCTGCTGCCGGCTCTGCGGCCTCTTCCGCGTCTTCGCCTTCGCCCTCAGACGAG 7800
     >>>>>>>>>>>>>>>>>>>>>>>>>>>>>>>>>>>>>>>>>>>>>>>>>>>>>>>>>>>>>>>>>>>>>>>>>>>>>>>>>>>>>>>>>>>>>>>>>>>>
     WPRE

7801 TCGGATCTCCCTTTGGGCCGCCTCCCCGCCTGGAATTCGAGCTCGGTACCTTTAAGACCAATGACTTACAAGGCAGCTGTAGATCTTAGCCACTTTTTAA 7900
                                                                                                       >>
                                                                                                       U3PPT
     >>>>>>>>>>>>>>>>>>
     WPRE

7901 AAGAAAAGGGGGGACTGGAAGGGCTAATTCACTCCCAACGAAGACAAGATCTGCTTTTTGCTTGTACTGGGTCTCTCTGGTTAGACCAGATCTGAGCCTG 8000
     >>>>>>>>>>>>>>>>>>>>                                                 >>>>>>
     U3PPT                                                                BsaI site
                                                                         >>>>>>>>>>>>>>>>>>>>>>>>>>>>>>>>
                                                                         HIV-1 5'LTR

8001 GGAGCTCTCTGGCTAACTAGGGAACCCACTGCTTAAGCCTCAATAAAGCTTGCCTTGAGTGCTTCAAGTAGTGTGTGCCCGTCTGTTGTGTGACTCTGGT 8100
     >>>>>>>>>>>>>>>>>>>>>>>>>>>>>>>>>>>>>>>>>>>>>>>>>>>>>>>>>>>>>>>>>>>>>>>>>>>>>>>>>>>>>>>>>>>>>>>>>>>>
     HIV-1 5'LTR

8101 AACTAGAGATCCCTCAGACCCTTTTAGTCAGTGTGGAAAATCTCTAGCAGTAGTAGTTCATGTCATCTTATTATTCAGTATTTATAACTTGCAAAGAAAT 8200
     >>>>>>>>>>>>>>>>>>>>>>>>>>>>>>>>>>>>>>>>>>>>>>>>
     HIV-1 5'LTR

8201 GAATATCAGAGAGTGAGAGGAACTTGTTTATTGCAGCTTATAATGGTTACAAATAAAGCAATAGCATCACAAATTTCACAAATAAAGCATTTTTTTCACT 8300
```

```
              *         *         *         *         *         *         *         *         *         *
8301 GCATTCTAGTTGTGGTTTGTCCAAACTCATCAATGTATCTTATCATGTCTGGCTCTAGCTATCCCGCCCCTAACTCCGCCCAGTTCCGCCCATTCTCCGC 8400
                                                                 >>>>>>>>>>>>>>>>>>>>>>>>>>>>>>>>>>>>>>>>
                                                                 SV40 ori

                                                                                                   8500
              *         *         *         *         *         *         *         *         *         *
8401 CCCATGGCTGACTAATTTTTTTTATTTATGCAGAGGCCGAGGCCGCCTCGGCCTCTGAGCTATTCCAGAAGTAGTGAGGAGGCTTTTTTGGAGGCCTAGG 8500
     >>>>>>>>>>>>>>>>>>>>>>>>>>>>>>>>>>>>>>
     SV40 ori

                                                                                                   8600
              *         *         *         *         *         *         *         *         *         *
8501 CTTTTGCGTCGAGACGTACCCAATTCGCCCTATAGTGAGTCGTATTACGCGCGCTCACTGGCCGTCGTTTTACAACGTCGTGACTGGGAAAACCCTGGCG 8600
                                                             <<<<<<<<<<<<<<<<<<
                                                             M13-fwd
                                <<<<<<<<<<<<<<<<<<<<<
                                T7
               <<<<<<
               BsmBI site

                                                                                                   8700
              *         *         *         *         *         *         *         *         *         *
8601 TTACCCAACTTAATCGCCTTGCAGCACATCCCCCTTTCGCCAGCTGGCGTAATAGCGAAGAGGCCCGCACCGATCGCCCTTCCCAACAGTTGCGCAGCCT 8700
                                                 W  R  N  S  E  E  A  R  T  D  R  P  S  Q  Q  L  R  S  L
                                                 >>>>>>>>>>>>>>>>>>>>>>>>>>>>>>>>>>>>>>>>>>>>>>>>>>>>>>>>
                                                 LacZ alpha

                                                                                                   8800
              *         *         *         *         *         *         *         *         *         *
8701 GAATGGCGAATGGCGCGACGCGCCCTGTAGCGGCGCATTAAGCGCGGCGGGTGTGGTGGTTACGCGCAGCGTGACCGCTACACTTGCCAGCGCCCTAGCG 8800
                                       >>>>>>>>>>>>>>>>>>>>>>>>>>>>>>>>>>>>>>>>>>>>>>>>>>>>>>>>>>>>>>>>>>
                                       F1 ori
                      >>>>>>>>>>>>>>>>>>>>>>>>>>>>>>>>>>>>>>>>>>>>>>>>>>>>>>>>>>>>>>>>>>>>>>>>>>>>>>>>>>>
                      M13 origin
      N  G  E  W
     >>>>>>>>>>>>>
     LacZ alpha

                                                                                                   8900
              *         *         *         *         *         *         *         *         *         *
8801 CCCGCTCCTTTCGCTTTCTTCCCTTCCTTTCTCGCCACGTTCGCCGGCTTTCCCCGTCAAGCTCTAAATCGGGGGCTCCCTTTAGGGTTCCGATTTAGTG 8900
     >>>>>>>>>>>>>>>>>>>>>>>>>>>>>>>>>>>>>>>>>>>>>>>>>>>>>>>>>>>>>>>>>>>>>>>>>>>>>>>>>>>>>>>>>>>>>>>>>>>>
     F1 ori
     >>>>>>>>>>>>>>>>>>>>>>>>>>>>>>>>>>>>>>>>>>>>>>>>>>>>>>>>>>>>>>>>>>>>>>>>>>>>>>>>>>>>>>>>>>>>>>>>>>>>
     M13 origin

                                                                                                   9000
              *         *         *         *         *         *         *         *         *         *
8901 CTTTACGGCACCTCGACCCCAAAAAACTTGATTAGGGTGATGGTTCACGTAGTGGGCCATCGCCCTGATAGACGGTTTTTCGCCCTTTGACGTTGGAGTC 9000
     >>>>>>>>>>>>>>>>>>>>>>>>>>>>>>>>>>>>>>>>>>>>>>>>>>>>>>>>>>>>>>>>>>>>>>>>>>>>>>>>>>>>>>>>>>>>>>>>>>>>
     F1 ori
     >>>>>>>>>>>>>>>>>>>>>>>>>>>>>>>>>>>>>>>>>>>>>>>>>>>>>>>>>>>>>>>>>>>>>>>>>>>>>>>>>>>>>>>>>>>>>>>>>>>>
     M13 origin

                                                                                                   9100
              *         *         *         *         *         *         *         *         *         *
9001 CACGTTCTTTAATAGTGGACTCTTGTTCCAAACTGGAACAACACTCAACCCTATCTCGGTCTATTCTTTTGATTTATAAGGGATTTTGCCGATTTCGGCC 9100
     >>>>>>>>>>>>>>>>>>>>>>>>>>>>>>>>>>>>>>>>>
     F1 ori
     >>>>>>>>>>>>>>>>>>>>>>>>>>>>>>>>>>>>>>>>>>>>>>>>>>>>>>>>>>>>>>>>>>>>>>>>>>>>>>>>>>>>>>>>>>>>>>>>>>>>
     M13 origin

                                                                                                   9200
              *         *         *         *         *         *         *         *         *         *
9101 TATTGGTTAAAAAATGAGCTGATTTAACAAAAATTTAACGCGAATTTTAACAAAATATTAACGTTTACAATTTCCCAGGTGGCACTTTTCGGGGAAATGT 9200
     >>>>>>>>>>>>>>>>>>>>>>>>>>>>>>>>>>>>>>>>>>>>>>>>>>>>>>>>>>>>>>>>>>>>>>>>>
     M13 origin

                                                                                                   9300
              *         *         *         *         *         *         *         *         *         *
9201 GCGCGGAACCCCTATTTGTTTATTTTTCTAAATACATTCAAATATGTATCCGCTCATGAGACAATAACCCTGATAAATGCTTCAATAATATTGAAAAAGG 9300
```

```
                                                                                                        9400
                          *         *         *         *         *         *         *         *         *         *
 9301 AAGAGTATGAGTATTCAACATTTCCGTGTCGCCCTTATTCCCTTTTTTGCGGCATTTTGCCTTCCTGTTTTTGCTCACCCAGAAACGCTGGTGAAAGTAA 9400

                                                                                                        9500
                          *         *         *         *         *         *         *         *         *         *
 9401 AAGATGCTGAAGATCAGTTGGGTGCACGAGTGGGTTACATCGAACTGGATCTCAACAGCGGTAAGATCCTTGAGAGTTTTCGCCCCGAAGAACGTTTTCC 9500

                                                                                                        9600
                          *         *         *         *         *         *         *         *         *         *
 9501 AATGATGAGCACTTTTAAAGTTCTGCTATGTGGCGCGGTATTATCCCGTATTGACGCCGGGCAAGAGCAACTCGGTCGCCGCATACACTATTCTCAGAAT 9600
          M  S  T  F  K  V  L  L  C  G  A  V  L  S  R  I  D  A  G  Q  E  Q  L  G  R  R  I  H  Y  S  Q  N
          >>>>>>>>>>>>>>>>>>>>>>>>>>>>>>>>>>>>>>>>>>>>>>>>>>>>>>>>>>>>>>>>>>>>>>>>>>>>>>>>>>>>>>>>>>>>>
          AmpR

                                                                                                        9700
                          *         *         *         *         *         *         *         *         *         *
 9601 GACTTGGTTGAGTACTCACCAGTCACAGAAAAGCATCTTACGGATGGCATGACAGTAAGAGAATTATGCAGTGCTGCCATAACCATGAGTGATAACACTG 9700
      D  L  V  E  Y  S  P  V  T  E  K  H  L  T  D  G  M  T  V  R  E  L  C  S  A  A  I  T  M  S  D  N  T  A
      >>>>>>>>>>>>>>>>>>>>>>>>>>>>>>>>>>>>>>>>>>>>>>>>>>>>>>>>>>>>>>>>>>>>>>>>>>>>>>>>>>>>>>>>>>>>>>>>>
      AmpR

                                                                                                        9800
                          *         *         *         *         *         *         *         *         *         *
 9701 CGGCCAACTTACTTCTGACAACGATCGGAGGACCGAAGGAGCTAACCGCTTTTTTGCACAACATGGGGGATCATGTAACTCGCCTTGATCGTTGGGAACC 9800
        A  N  L  L  L  T  T  I  G  G  P  K  E  L  T  A  F  L  H  N  M  G  D  H  V  T  R  L  D  R  W  E  P
      >>>>>>>>>>>>>>>>>>>>>>>>>>>>>>>>>>>>>>>>>>>>>>>>>>>>>>>>>>>>>>>>>>>>>>>>>>>>>>>>>>>>>>>>>>>>>>>>>
      AmpR

                                                                                                        9900
                          *         *         *         *         *         *         *         *         *         *
 9801 GGAGCTGAATGAAGCCATACCAAACGACGAGCGTGACACCACGATGCCTGTAGCAATGGCAACAACGTTGCGCAAACTATTAACTGGCGAACTACTTACT 9900
       E  L  N  E  A  I  P  N  D  E  R  D  T  T  M  P  V  A  M  A  T  T  L  R  K  L  L  T  G  E  L  L  T
      >>>>>>>>>>>>>>>>>>>>>>>>>>>>>>>>>>>>>>>>>>>>>>>>>>>>>>>>>>>>>>>>>>>>>>>>>>>>>>>>>>>>>>>>>>>>>>>>>
      AmpR

                                                                                                        10000
                          *         *         *         *         *         *         *         *         *         *
 9901 CTAGCTTCCCGGCAACAATTAATAGACTGGATGGAGGCGGATAAAGTTGCAGGACCACTTCTGCGCTCGGCCCTTCCGGCTGGCTGGTTTATTGCTGATA 10000
      L  A  S  R  Q  Q  L  I  D  W  M  E  A  D  K  V  A  G  P  L  L  R  S  A  L  P  A  G  W  F  I  A  D  K
      >>>>>>>>>>>>>>>>>>>>>>>>>>>>>>>>>>>>>>>>>>>>>>>>>>>>>>>>>>>>>>>>>>>>>>>>>>>>>>>>>>>>>>>>>>>>>>>>>
      AmpR

                                                                                                        10100
                          *         *         *         *         *         *         *         *         *         *
10001 AATCTGGAGCCGGTGAGCGTGGGTCTCGCGGTATCATTGCAGCACTGGGGCCAGATGGTAAGCCCTCCCGTATCGTAGTTATCTACACGACGGGGAGTCA 10100
        S  G  A  G  E  R  G  S  R  G  I  I  A  A  L  G  P  D  G  K  P  S  R  I  V  V  I  Y  T  T  G  S  Q
      >>>>>>>>>>>>>>>>>>>>>>>>>>>>>>>>>>>>>>>>>>>>>>>>>>>>>>>>>>>>>>>>>>>>>>>>>>>>>>>>>>>>>>>>>>>>>>>>>
      AmpR
                           >>>>>>
                           BsaI site

                                                                                                        10200
                          *         *         *         *         *         *         *         *         *         *
10101 GGCAACTATGGATGAACGAAATAGACAGATCGCTGAGATAGGTGCCTCACTGATTAAGCATTGGTAACTGTCAGACCAAGTTTACTCATATATACTTTAG 10200
       A  T  M  D  E  R  N  R  Q  I  A  E  I  G  A  S  L  I  K  H  W
      >>>>>>>>>>>>>>>>>>>>>>>>>>>>>>>>>>>>>>>>>>>>>>>>>>>>>>>>>>>>>>>>
      AmpR

                                                                                                        10300
                          *         *         *         *         *         *         *         *         *         *
10201 ATTGATTTAAAACTTCATTTTTAATTTAAAAGGATCTAGGTGAAGATCCTTTTTGATAATCTCATGACCAAAATCCCTTAACGTGAGTTTTCGTTCCACT 10300
                                                                   >>>>>>>>>>>>>>>>>>>>>>>>>>>>>>>>>>>>>>>
                                                                   ColE1 origin

                                                                                                        10400
                          *         *         *         *         *         *         *         *         *         *
10301 GAGCGTCAGACCCCGTAGAAAAGATCAAAGGATCTTCTTGAGATCCTTTTTTTCTGCGCGTAATCTGCTGCTTGCAAACAAAAAAACCACCGCTACCAGC 10400
      >>>>>>>>>>>>>>>>>>>>>>>>>>>>>>>>>>>>>>>>>>>>>>>>>>>>>>>>>>>>>>>>>>>>>>>>>>>>>>>>>>>>>>>>>>>>>>>>>
      ColE1 origin
```

```
                                                                                                           10500
             *         *         *         *         *         *         *         *         *         *
10401 GGTGGTTTGTTTGCCGGATCAAGAGCTACCAACTCTTTTTCCGAAGGTAACTGGCTTCAGCAGAGCGCAGATACCAAATACTGTCCTTCTAGTGTAGCCG 10500
      >>>>>>>>>>>>>>>>>>>>>>>>>>>>>>>>>>>>>>>>>>>>>>>>>>>>>>>>>>>>>>>>>>>>>>>>>>>>>>>>>>>>>>>>>>>>>>>>>>
      ColE1 origin

                                                                                                           10600
             *         *         *         *         *         *         *         *         *         *
10501 TAGTTAGGCCACCACTTCAAGAACTCTGTAGCACCGCCTACATACCTCGCTCTGCTAATCCTGTTACCAGTGGCTGCTGCCAGTGGCGATAAGTCGTGTC 10600
      >>>>>>>>>>>>>>>>>>>>>>>>>>>>>>>>>>>>>>>>>>>>>>>>>>>>>>>>>>>>>>>>>>>>>>>>>>>>>>>>>>>>>>>>>>>>>>>>>>
      ColE1 origin

                                                                                                           10700
             *         *         *         *         *         *         *         *         *         *
10601 TTACCGGGTTGGACTCAAGACGATAGTTACCGGATAAGGCGCAGCGGTCGGGCTGAACGGGGGGTTCGTGCACACAGCCCAGCTTGGAGCGAACGACCTA 10700
      >>>>>>>>>>>>>>>>>>>>>>>>>>>>>>>>>>>>>>>>>>>>>>>>>>>>>>>>>>>>>>>>>>>>>>>>>>>>>>>>>>>>>>>>>>>>>>>>>>
      ColE1 origin

                                                                                                           10800
             *         *         *         *         *         *         *         *         *         *
10701 CACCGAACTGAGATACCTACAGCGTGAGCTATGAGAAAGCGCCACGCTTCCCGAAGGGAGAAAGGCGGACAGGTATCCGGTAAGCGGCAGGGTCGGAACA 10800
      >>>>>>>>>>>>>>>>>>>>>>>>>>>>>>>>>>>>>>>>>>>>>>>>>>>>>>>>>>>>>>>>>>>>>>>>>>>>>>>>>>>>>>>>>>>>>>>>>>
      ColE1 origin

                                                                                                           10900
             *         *         *         *         *         *         *         *         *         *
10801 GGAGAGCGCACGAGGGAGCTTCCAGGGGGAAACGCCTGGTATCTTTATAGTCCTGTCGGGTTTCGCCACCTCTGACTTGAGCGTCGATTTTTGTGATGCT 10900
      >>>>>>>>>>>>>>>>>>>>>>>>>>>>>>>>>>>>>>>>>>>>>>>>>>>>>>>>>>>>>>>>>>>>>>>>>>>>>>>>>>>>>>>>>>>>>>>>>>
      ColE1 origin

                                                                                                           11000
             *         *         *         *         *         *         *         *         *         *
10901 CGTCAGGGGGGCGGAGCCTATGGAAAAACGCCAGCAACGCGGCCTTTTTACGGTTCCTGGCCTTTTGCTGGCCTTTTGCTCACATGTTCTTTCCTGCGTT 11000
      >>>>>>>>>>>>>>>>>>>>>>>>>>>>>>>>>>>>>>>>>>>>
      ColE1 origin

                                                                                                           11100
             *         *         *         *         *         *         *         *         *         *
11001 ATCCCCTGATTCTGTGGATAACCGTATTACCGCCTTTGAGTGAGCTGATACCGCTCGCCGCAGCCGAACGACCGAGCGCAGCGAGTCAGTGAGCGAGGAA 11100

                                                                                                           11200
             *         *         *         *         *         *         *         *         *         *
11101 GCGGAAGAGCGCCCAATACGCAAACCGCCTCTCCCCGCGCGTTGGCCGATTCATTAATGCAGCTGGCACGACAGGTTTCCCGACTGGAAAGCGGGCAGTG 11200

                                                                                                           11300
             *         *         *         *         *         *         *         *         *         *
11201 AGCGCAACGCAATTAATGTGAGTTAGCTCACTCATTAGGCACCCCAGGCTTTACACTTTATGCTTCCGGCTCGTATGTTGTGTGGAATTGTGAGCGGATA 11300
                                                                                       >>>>>>>>>>>>>>>>>
                                                                                       LacO
```

Supplementary Figure 1. Annotated full DNA sequence of the pRRL.sin.CRISPR/Cas9 plasmid..
